# Cox Proportional Hazard Regression Versus a Deep Learning Algorithm in the Prediction of Dementia: An Analysis Based on Periodic Health Examination

**DOI:** 10.2196/13139

**Published:** 2019-08-30

**Authors:** Woo Jung Kim, Ji Min Sung, David Sung, Myeong-Hun Chae, Suk Kyoon An, Kee Namkoong, Eun Lee, Hyuk-Jae Chang

**Affiliations:** 1 Department of Psychiatry Myongji Hospital, Hanyang University College of Medicine Goyang Republic of Korea; 2 Institute of Behavioral Science in Medicine Yonsei University College of Medicine Seoul Republic of Korea; 3 Gyeonggi Provincial Dementia Center Suwon Republic of Korea; 4 Division of Cardiology Severance Cardiovascular Hospital, Yonsei University College of Medicine Seoul Republic of Korea; 5 Data Science Team kt NexR Seoul Republic of Korea; 6 AI R&D Lab of Selvas AI, Inc Seoul Republic of Korea; 7 Department of Psychiatry Yonsei University College of Medicine Seoul Republic of Korea; 8 Severance Biomedical Science Institute Yonsei University College of Medicine Seoul Republic of Korea

**Keywords:** dementia, deep learning, proportional hazards models

## Abstract

**Background:**

With the increase in the world’s aging population, there is a growing need to prevent and predict dementia among the general population. The availability of national time-series health examination data in South Korea provides an opportunity to use deep learning algorithm, an artificial intelligence technology, to expedite the analysis of mass and sequential data.

**Objective:**

This study aimed to compare the discriminative accuracy between a time-series deep learning algorithm and conventional statistical methods to predict all-cause dementia and Alzheimer dementia using periodic health examination data.

**Methods:**

Diagnostic codes in medical claims data from a South Korean national health examination cohort were used to identify individuals who developed dementia or Alzheimer dementia over a 10-year period. As a result, 479,845 and 465,081 individuals, who were aged 40 to 79 years and without all-cause dementia and Alzheimer dementia, respectively, were identified at baseline. The performance of the following 3 models was compared with predictions of which individuals would develop either type of dementia: Cox proportional hazards model using only baseline data (HR-B), Cox proportional hazards model using repeated measurements (HR-R), and deep learning model using repeated measurements (DL-R).

**Results:**

The discrimination indices (95% CI) for the HR-B, HR-R, and DL-R models to predict all-cause dementia were 0.84 (0.83-0.85), 0.87 (0.86-0.88), and 0.90 (0.90-0.90), respectively, and those to predict Alzheimer dementia were 0.87 (0.86-0.88), 0.90 (0.88-0.91), and 0.91 (0.91-0.91), respectively. The DL-R model showed the best performance, followed by the HR-R model, in predicting both types of dementia. The DL-R model was superior to the HR-R model in all validation groups tested.

**Conclusions:**

A deep learning algorithm using time-series data can be an accurate and cost-effective method to predict dementia. A combination of deep learning and proportional hazards models might help to enhance prevention strategies for dementia.

## Introduction

### Background

The prevention of dementia is a public health challenge in countries with aging populations [1]. Systematic reviews and meta-analyses have shown that lifestyle and health conditions affect the incidence of dementia, including Alzheimer dementia [2-7]. In South Korea, a country with one of the fastest-growing elderly populations [8], the National Health Insurance Service (NHIS) runs periodic general health examination programs [9]. The large time-series health examination dataset established by the NHIS includes lifestyle information and the results of periodic routine medical examinations of a nationwide sample of the Korean population.

Medical time-series data are often analyzed using conventional statistical methods such as Cox proportional hazards regression models. In the field of computer science, machine learning can (semi)automatically classify mass data and thus has been applied to diagnose diseases and predict outcomes using large medical datasets [10-12]. Deep learning, a subfield of machine learning, has recently enabled powerful new analyses of time-series data [13,14]. Among the deep learning algorithms, the recurrent neural network (RNN) is considered the most suitable method for analyzing time-series data [15,16]. The RNN in its basic form has a *vanishing gradient* problem in the long-term learning process, however. The long short-term memory (LSTM) technique was developed to overcome that problem [17].

### Objective

The application of deep learning to predict disease using data from routine health examinations may lead to improvements in preventive medicine and early treatment. Until now, applications of deep learning algorithms to predict dementia have focused on neuroimaging data [18-20]. To our knowledge, there is no published research on the application of deep learning to analyze time-series health examination data. This study aimed to compare the accuracy of Cox proportional hazards regression models with that of LSTM in predicting all-cause dementia and Alzheimer dementia using the NHIS time-series health examination dataset.

## Methods

### Explanation of the Data

The NHIS provides health insurance to the entire population of South Korea and stores medical and prescription records for billing purposes. To serve academic interests, the NHIS also develops research databases, including the NHIS-Health Screening Cohort (NHIS-HEALS). The method of data construction for the NHIS-HEALS is the same as that for another cohort, the NHIS-National Sample Cohort [21,22]. The NHIS-HEALS dataset contains information on more than 500,000 randomly sampled individuals nationwide who attended NHIS periodic general health examinations, representing 10% of the entire Korean population who underwent a baseline medical examination between 2002 and 2003 and routine follow-up examinations every 2 years until 2013. The NHIS-HEALS cohort can be considered to reflect the Korean adult population (aged 40-79 years) because every Korean older than 40 years is recommended to have a routine health examination biennially. The baseline for the NHIS-HEALS dataset is defined as the years 2002 to 2003 [23].

The NHIS-HEALS dataset incorporates several databases. We used the health examination database, the health care utilization database, and the eligibility database [23]. The health examination database contains physical measurements such as height, weight, and blood pressure; data from blood tests including fasting glucose, lipid profile, liver panel, and hemoglobin; and the results of urinalysis and self-reported questionnaires about lifestyle and family and personal medical histories. The health care utilization database includes medical claims data on inpatient and outpatient health care services including diagnoses, diagnostic tests, therapeutic procedures, length of hospital stay, and prescribed medications and dosages. The eligibility database has information on demographic factors, economic status, insurance eligibility, and cause of death.

As the NHIS-HEALS dataset is representative of the entire Korean population and contains a huge amount of time-series data, including health examination results, insurance eligibility, and health care utilization, it can be used to assess the accuracy of different predictive models of disease incidence. We used the NHIS-HEALS dataset to compare the effectiveness of an LSTM deep learning algorithm with that of conventional statistical methods to predict all-cause dementia and Alzheimer dementia. This study was approved by the institutional review board of Yonsei University, Severance Hospital, Seoul, South Korea (IRB no 4-2016-0383).

### Study Population and Sample Selection

We used the diagnostic codes in the health care utilization database as outcome variables and risk factors extracted from the health examination database as independent variables. Even if an individual had only 2 health examination records in the health examination database, we determined whether or not that individual had been diagnosed with dementia by searching the information in the health care utilization database up until the last date for which records were stored in the NHIS-HEALS (December 31, 2013).

The primary outcomes in our analyses were instances of all-cause dementia (F00.X, F01.X, F02.X, F03.X, and G30.X) and Alzheimer dementia (F00.X and G30.X), which we identified using medical diagnostic codes according to the International Classification of Diseases, 10th edition. Only the individuals with the diagnostic codes to have developed dementia were considered. Individuals who died or were lost to follow-up without a diagnosis of dementia were considered to not have suffered a dementia event. The time to event was defined as the time between the date of the first health examination and that of the first diagnosis of dementia or the most recent follow-up.

**Figure 1:**
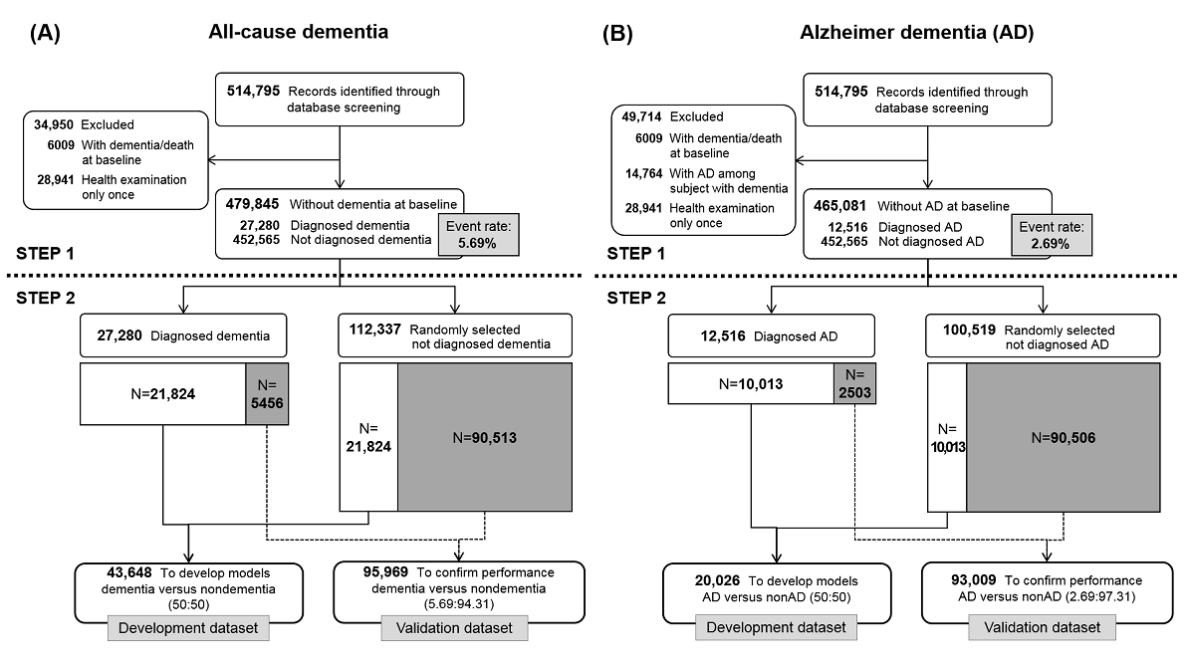
Study design and sample selection. (A) All-cause dementia; (B) Alzheimer dementia.

Figure 1 shows the sample selection process in 2 steps (with all-cause dementia and Alzheimer dementia separately labeled as *A* and *B*). The first step describes the selection of candidate individuals from the NHIS-HEALS cohort whose data could be used for predictive modeling. The second step describes the process of dividing the data into development and validation datasets for machine learning. The development datasets were used to fit the parameters of classifiers (ie, criteria that helped to discriminate individuals who developed dementia during the study period from those who did not develop dementia) in each model. The validation datasets were used to assess the generalization error of the final models.

To create the development and validation datasets for all-cause dementia and Alzheimer dementia, we first identified 514,795 individuals with records of a health examination in the baseline year (2002-2003). To analyze all-cause dementia, we excluded individuals with records of all-cause dementia or death at baseline and those with no further health examinations after the baseline year. Of the remaining 479,845 individuals, 27,280 developed all-cause dementia during the study period, resulting in an event rate of 5.69% (Figure 1). We applied the same procedure to analyze Alzheimer dementia among 465,081 individuals and found an event rate of 2.69% (Figure 1).

The deep learning method has the advantage that it can identify patterns in each outcome (eg, yes or no; or event or nonevent). Deep learning is considered to have high predictive accuracy in classification studies; however, an extremely imbalanced dataset can pose a challenge to the detection of patterns in outcome variables. The fundamental cause of that problem is that smaller amount of data provides less concrete evidence for specific patterns than larger amounts of data. Thus, we attempted to deal with this limitation by generating 1:1 allocation through undersampling, which has been used in previous studies [24,25]. To build a precise and predictive deep learning model, we used undersampling to adjust the imbalance between the number of dementia cases and the number of nondementia cases in the development datasets, resulting in a more precise and predictive deep learning model. The numbers of cases in the validation datasets still reflected the actual event rates in the NHIS-HEALS cohort.

To finish the construction of the development and validation datasets for all-cause dementia, we divided the 27,280 individuals who developed all-cause dementia into 2 datasets with a size ratio of 8:2, corresponding to the development and validation datasets. The development dataset of 43,648 individuals consisted of 21,824 with dementia (80.00% of 27,280 individuals with dementia) and 21,824 without dementia as a 1:1 ratio to solve the imbalance problem in classification. The validation dataset included 5456 individuals who developed all-cause dementia (20.00% of 27,280 who developed all-cause dementia) along with 90,513 randomly selected individuals who did not develop all-cause dementia, for a total of 95,969 individuals. In the development dataset, there were 946 deaths (4.30%) among the 21,824 individuals who did not develop all-cause dementia. In the validation dataset, there were 3905 deaths (4.30%) among the 90,513 individuals who did not develop all-cause dementia. Thus, the event rates of all-cause dementia in the development and validation datasets were 50.00% and 5.69%, respectively.

We constructed the development and validation datasets for Alzheimer dementia by the same process. The event rates of Alzheimer dementia in the development and validation datasets were 50.00% (n=20,026) and 2.69% (n=93,009), respectively. Secondary analyses by age group are presented in Multimedia Appendix 1.

### Measures

We used the following health examination variables from the NHIS-HEALS dataset in our deep learning and Cox proportional hazards models: age, sex, body mass index (BMI), systolic blood pressure (SBP), diastolic blood pressure (DBP), fasting blood glucose, total cholesterol, current smoking status, exercise status, and past medical history (ie, cardiovascular disease, diabetes, and hypertension from a self-reported questionnaire and psychiatric disorders [F04-F09, F20-F29, F30-F39, F70-F79, and F80-F89] and neurological disorders [G00-G09, G10-G14, G20-G26, G31-G39, and G40-G47] from diagnostic codes). All those variables were previously reported as risk factors for dementia [1-7] and were chosen for inclusion in our models by expert geriatric psychiatrists (WJK, SKA, KN, and EL) after review and discussion. Data were missing for approximately 4% of the included individuals. We used the multiple imputation (MI) method to deal with missing data; each missing data point was managed by the fully conditional specification (FCS) regression and FCS discriminant function proposed by the SAS MI procedure (SAS Inc) [26,27].

### Statistical and Deep Learning Predictive Models

We created 3 models to predict dementia based on Cox proportional hazards regression and deep learning. First, we used Cox proportional hazards regression to develop 2 predictive models [28]: a model with baseline data only (HR-B) and a model with repeated measurements (HR-R). The HR-B model used risk factor data obtained from the first screening examination. The HR-R model used the mean, minimum, maximum, and standard deviation (SD) values of continuous variables and the mean and SD values of categorical variables recorded in multiple health examinations over the study period. As in a previous study of cumulative information using the Cox algorithms [28], we intended to show how much the change in risk factors affects the occurrence of dementia. The hazard ratio of each risk factor can be estimated according to the changes of each information based on the value of minimum, maximum, or SD. For example, if the coefficient value of SD of BMI was large, a larger change of BMI in the observation period meant that it affected the occurrence of dementia. Values for continuous variables were calculated at the individual level. We assigned values of *0* or *1* to categorical variables such as smoking status, exercise status, and past medical history. The mean value was coded as 0 when it was less than 0.5, and 1 if otherwise in the HR-R model. As the categorical variables were collected from self-reported questionnaires, we considered that some data points may be missing because of possible mistakes in self-report.

Similar to a previous study that applied Cox algorithms to cumulative information [28], we intended to determine the extent to which changes in the risk factors affect the occurrence of dementia. The hazard ratio of each risk factor in the HR-R model can be estimated according to the minimum, maximum, or SD of the values of the risk factors. For example, if the coefficient of the SD of BMI is large, then a large change of BMI in the observation period would be determined to have affected the occurrence of dementia. The Cox proportional hazards regression model can be written as follows:

*h(t)=h*_0_*(t)exp(b*_1_*X*_1_*+ b*_2_*X*_2_*+ b*_3_*X*_3_*+...+ b*_p_*X*_p_*)*

where *h(t)* is the expected hazard at time *t*, and *h*_0_*(t)* is the baseline hazard, representing the hazard when all of the predictors *X*_1_, *X*_2__,_...*X*_p_ are equal to zero. The predicted hazard *h(t)* is the product of the baseline hazard *h*_0_*(t)* and the exponential function of the linear combination of the predictors. Thus, the predictors have a multiplicative or proportional effect on the predicted hazard.

Flexible models, such as neural networks, have the potential to discover unanticipated features that are missed by conventional statistical models. We developed a deep learning model based on RNN-LSTM to overcome problems in the original RNN algorithm. Although the RNN is a simple and powerful model, it is difficult to train appropriately because of the vanishing gradient problem [29]. Unlike the feedforward neural network, where the input and output are in only 1 direction, the RNN is a neural network with a recursive connectivity structure that reflects the output of the previous input to the next input. This characteristic is an advantage of the RNN, which learns the time continuity and dependent relationships of time-series data such as voice, text, and signal. However, a simple architecture in which input is fed back to the output has the problem that normal learning is difficult because of the rapidly diminishing or increasing influence of the previous input. That is, it is impossible for the model to learn correlations between distant events when long-term components decrease exponentially to zero. In other words, the basic cyclic structure of the RNN causes the model to lose accumulated information as the length of the continuous input increases in the learning process (eg, multiple time steps such as repeated health examinations), which is a problem for parameter estimation. To address this *vanishing* problem, LSTM is designed to extend the structure of the neurons into memory blocks so that the memory cell within each node can properly adjust the effect of previous inputs during the learning process [13,14,30,31]. The iconography of each type of neural network is shown in Multimedia Appendix 2.

**Figure 2:**
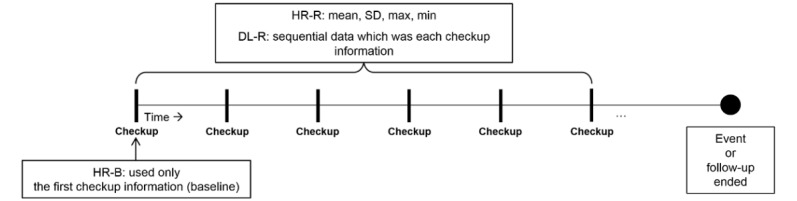
Conceptual diagram showing longitudinal data collection. DL-R: deep learning model with repeated measurements; HR-B: hazards regression model with baseline data only; HR-R: hazards regression model with repeated measurements; max: maximum; min: minimum.

We used the LSTM algorithm suggested by Hochreiter and Schmidhuber [17] to solve the long-term dependency problem and increase the learning ability of our deep learning model. As our data consisted of multiple time steps, the RNN-LSTM algorithm allowed us to avoid learning deficits because of the vanishing gradient problem [13,14,30,31]. In the RNN-LSTM model, the importance of each variable was trained during the deep learning process; features with missing values were included, and specific feature selection was not executed. We applied a single hidden layer consisting of 64 LSTM cells. As a regularization technique, we applied a 0.5 dropout probability [32]. We used Xavier initialization to initialize all the weights [33]. To optimize the parameters of the algorithm, we used root mean square propagation [34]. We applied a learning rate of 0.001 and a momentum of 0.9. We applied an early stopping technique to avoid overfitting the learning data with model performance [35]. The DL-R model used all the variables included in the HR-R model. As the RNN-LSTM model is specialized for the analysis of time-series data, we developed its algorithm using raw NHIS-HEALS data from medical examinations instead of descriptive statistics. Additional details of the RNN-LSTM model, including its construction and algorithm development, are shown in Multimedia Appendix 3. The 3 different models (HR-B, HR-R, and DL-R) used in this study are depicted in Figure 2. Details of the variables used in each model are described in Multimedia Appendix 4.

We compared the performance among the 3 predictive models. For the Cox hazards regression models (HR-B and HR-R), we presented the performance results using C-statistics. For the deep learning RNN-LSTM model (DL-R), we presented the performance results using the area under the receiver operating characteristic curve (AUC), which corresponds to the C-statistic in hazards regression analysis [36].

We calculated the integrated discrimination improvement and net reclassification improvement (NRI) to determine whether the DL-R model had an advantage in discrimination and reclassification over the HR-R model. To calculate the NRI, we divided the samples into 2 groups based on the risk for all-cause dementia or Alzheimer dementia, with the cutoff between the 2 groups set at 50% risk (ie, ≥50% and <50%).

As age is an important risk factor for dementia, we performed secondary analyses with the study population stratified by age (40-59 years and 60-79 years) to improve the predictive performance of the models. In South Korea, people aged 60 years or older are considered to be at high risk for developing dementia and are included in a national dementia screening and management program. We wanted to compare the performances of the predictive models for individuals younger and older than that age. We used the same procedures and methods to analyze the main groups and stratified groups.

**Figure 3:**
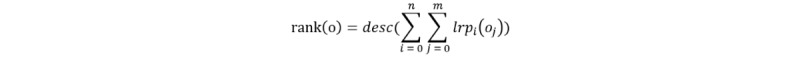
Expression of the ranking of risk factors.

In addition, to better understand the results of the deep learning model, we ranked the influence of the risk factors using layerwise relevance propagation (LRP), which is one of the explainable artificial intelligence techniques [37] used in artificial neural networks. [38,39]. The LRP values for each sample were summed and sorted in descending order. The ranking of the risk factors was expressed in Figure 3.

We used an Intel Core i7-4790 3.60 GHz processor, 16 GB memory, and an Nvidia GTX TITAN X 1 GHz graphics processor to develop and run the models. For the development of the DL-R model, we used Python 3.5 (programming language) and TensorFlow 1.3 (framework). TensorFlow is an open-source machine learning framework with source code and algorithms that have been shown to be stable by a broad range of feedback from users. We conducted all statistical analyses using SAS version 9.4 (SAS Inc) and R (www.R-project.org) software. For the data selection and imputation, we used the MI procedure in SAS (SAS Inc). For modeling, we used the R packages sas7bdat, survival, and MASS.

## Results

**Figure 4:**
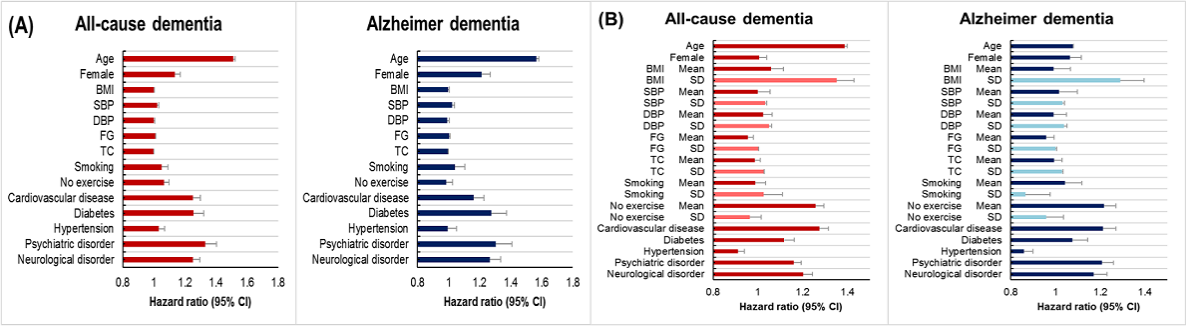
Summary of hazard ratios and 95% confidence intervals in the hazards regression models (40-79 years of age). BMI: body mass index; DBP: diastolic blood pressure; FG: fasting plasma glucose; SBP: systolic blood pressure; TC: total cholesterol.

Table 1 shows the baseline characteristics of the development datasets and the means and SDs of the repeated measurements in the development datasets for the HR-R model. The characteristics of the validation datasets, including the event rates of dementia, are shown in Table 2. The hazard ratios used to build the HR-B and HR-R models are shown in Figure 4. The HR-B models for both all-cause dementia and Alzheimer dementia identified the following risk factors: age, female sex, SBP, fasting glucose, cardiovascular disease, diabetes, psychiatric disorder, and neurological disorder (see Multimedia Appendix 5). The risk factors identified by the HR-R models for both all-cause dementia and Alzheimer dementia were age, female sex, no exercise, cardiovascular disease, diabetes, psychiatric disorder, and neurological disorder. In addition, the SDs of BMI, SBP, DBP, fasting glucose, and total cholesterol were significant predictors of both all-cause dementia and Alzheimer dementia. The details of secondary analyses are presented in Multimedia Appendix 6. Multimedia Appendix 7 shows the ranking of the risk factors in the DL-R model. For all-cause dementia among individuals aged 40 to 79 years, the risk factors were ranked in the following order, from the strongest effect to the weakest effect: sex, age, exercise, smoking, and cardiovascular disease. Among individuals aged 60 to 79 years who developed Alzheimer dementia, sex was the highest-ranked risk factor, and age had the lowest rank.

**Table 1:** Characteristics of the development datasets from the National Health Insurance Service-Health Screening Cohort (40-79 years of age).

Variable	All-cause dementia (n=43,648)		Alzheimer dementia (n=20,026)	
	Baseline	Repeated measurement	Baseline	Repeated measurement
Duration of follow-up (years), mean (SD)	9.11 (2.27)	—^a^	9.20 (2.21)	—
Number of periodic health examinations (n), mean (SD)	4.72 (2.34)	—	4.76 (2.33)	—
Age (years), mean (SD)	57.97 (10.61)	—	58.34 (10.70)	—
Sex (female), n (%)	22,374 (51.26)	—	10,465 (52.26)	—
Body mass index (kg/m^2^), mean (SD)	23.98 (3.06)	23.90 (2.89)	23.95 (3.09)	23.86 (2.92)
Systolic blood pressure (mm Hg), mean (SD)	129.45 (18.85)	128.64 (13.34)	129.17 (18.74)	128.45 (13.20)
Diastolic blood pressure (mm Hg), mean (SD)	80.16 (11.81)	79.04 (7.85)	79.85 (11.76)	78.83 (7.75)
Fasting plasma glucose (mg/dL), mean (SD)	100.56 (38.08)	102.17 (26.46)	100.54 (38.82)	102.03 (25.96)
Total cholesterol (mg/dL), mean (SD)	201.79 (39.14)	199.82 (30.70)	201.64 (38.67)	199.64 (30.75)
Smoking, n (%)	9192 (21.06)	8302 (19.02)	4088 (20.41)	3667 (18.31)
No exercise, n (%)	16,492 (37.78)	24,530 (56.20)	7522 (37.56)	11,233 (56.09)
Cardiovascular disease, n (%)	5061 (11.60)	23,463 (53.76)	2273 (11.35)	10,516 (52.51)
Diabetes, n (%)	2708 (6.20)	7092 (16.25)	1271 (6.35)	3317 (16.56)
Hypertension, n (%)	5447 (12.48)	17,517 (40.13)	2442 (12.19)	7919 (39.54)
Psychiatric disorder, n (%)	2308 (5.29)	17,088 (39.15)	1064 (5.31)	7941 (39.65)
Neurological disorder, n (%)	5920 (13.56)	28,105 (64.39)	2720 (13.58)	12,854 (64.19)

^a^Not applicable.

**Table 2:** Characteristics of the validation datasets from the National Health Insurance Service-Health Screening Cohort (40-79 years of age).

Variable	All-cause dementia (n=95,969)		Alzheimer dementia (n=93,009)	
	Baseline	Repeated measurement	Baseline	Repeated measurement
Duration of follow-up (years), mean (SD)	10.39 (1.44)	—^a^	10.48 (1.31)	—
Number of periodic health examinations, mean (SD)	5.66 (2.56)	—	5.71 (2.56)	—
Age (years), mean (SD)	52.53 (9.35)	—	52.22 (9.17)	—
Sex (female), n (%)	43,786 (45.63)	—	42,178 (45.35)	—
Body mass index (kg/m^2^), mean (SD)	24.04 (2.96)	24.02 (2.80)	24.04 (2.96)	24.02 (2.80)
Systolic blood pressure (mm Hg), mean (SD)	126.80 (18.06)	126.34 (12.45)	126.68 (18.00)	126.19 (12.38)
Diastolic blood pressure (mm Hg), mean (SD)	79.51 (11.67)	78.49 (7.60)	79.50 (11.66)	78.44 (7.58)
Fasting plasma glucose (mg/dL), mean (SD)	97.76 (33.23)	100.02 (22.11)	97.65 (33.06)	99.96 (22.07)
Total cholesterol (mg/dL), mean (SD)	200.43 (38.45)	199.26 (29.48)	200.35 (38.34)	199.25 (29.32)
Smoking, n (%)	23,104 (24.07)	20,438 (21.30)	22,593 (24.29)	19,966 (21.47)
No exercise, n (%)	41,179 (42.91)	62,805 (65.44)	40,125 (43.14)	61,415 (66.03)
Cardiovascular disease, n (%)	6,629 (6.91)	39,848 (41.52)	6,188 (6.65)	38,004 (40.86)
Diabetes, n (%)	3744 (3.90)	13,116 (13.67)	3472 (3.73)	12,537 (13.48)
Hypertension, n (%)	7609 (7.93)	34,033 (35.46)	7120 (7.66)	32,720 (35.18)
Psychiatric disorder, n (%)	3209 (3.34)	28,723 (29.93)	2992 (3.22)	27,321 (29.37)
Neurological disorder, n (%)	8755 (9.12)	52,773 (54.99)	8283 (8.91)	50,572 (54.37)
Event rate, n (%)	5456 (5.69)	5456 (5.69)	2503 (2.69)	2503 (2.69)

^a^Not applicable.

The performance of the models is shown in Table 3. The discrimination indices (with 95% CIs) for the HR-B, HR-R, and DL-R models to predict all-cause dementia among individuals aged 40 to 79 years in the validation datasets were 0.84 (0.83-0.85), 0.87 (0.86-0.88), and 0.90 (0.90-0.90), respectively, indicating that the DL-R model performed the best, and the HR-R model performed better than the HR-B model. The discrimination indices for the HR-B, HR-R, and DL-R models to predict Alzheimer dementia among individuals aged 40 to 79 years in the validation datasets were 0.87 (0.86-0.88), 0.90 (0.88-0.91), and 0.91 (0.91-0.91), respectively, again indicating that the DL-R model performed the best, and the HR-R model performed better than the HR-B model. All the models performed better for Alzheimer dementia than for all-cause dementia. The results of secondary analyses by age group were similar to those of the main analyses; the predictive performance for Alzheimer dementia was better than that for all-cause dementia (see Multimedia Appendix 8).

**Figure 5:**
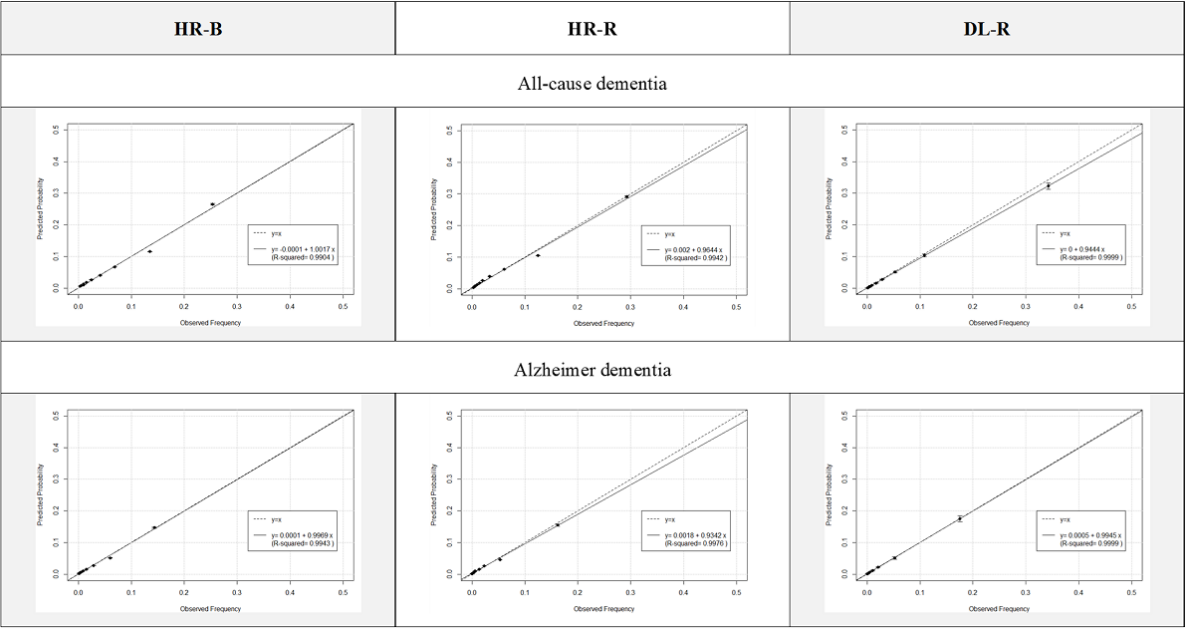
Calibration plots for each model (40-79 years of age). DL-R: deep learning model with repeated measurements; HR-B: hazards regression model with baseline data only; HR-R: hazards regression model with repeated measurements.

A comparison of the performance between the HR-R and DL-R models is shown in Table 4. The DL-R model demonstrated better AUCs than the HR-R model for both all-cause dementia (difference 0.034, 95% CI 0.029-0.039; *P*<.001) and Alzheimer dementia (difference 0.024, 95% CI 0.018-0.031; *P*<.001; see Multimedia Appendix 9). Calibration plots for the DL-R and HR-R models are shown in Figure 5. Calibration plots for each model by age group are shown in Multimedia Appendix 10.

**Table 3:** Comparison of the models’ performance to predict all-cause dementia and Alzheimer dementia in individuals aged 40 to 79 years.

Performance variable	All-cause dementia^a,b^			Alzheimer dementia^a,b^		
	HR-B^c^	HR-R^d^	DL-R^e^	HR-B	HR-R	DL-R
Discrimination (performance)	0.84 (0.83-0.85)	0.87 (0.86-0.88)	0.90 (0.90-0.90)	0.87 (0.86-0.88)	0.90 (0.88-0.91)	0.91 (0.91-0.91)
Sensitivity (%)	80.41 (79.35-81.46)	80.17 (79.11-81.23)	83.50 (82.52-84.49)	82.90 (81.43-84.38)	80.54 (78.99-82.09)	87.62 (86.32-88.91)
Specificity (%)	73.23 (72.94-73.52)	77.88 (77.61-78.15)	79.88 (79.61-80.14)	75.86 (75.58-76.13)	81.25 (80.99-81.5)	78.66 (78.40-78.93)
Accuracy (%)	73.64 (73.36-73.92)	78.01 (77.75-78.27)	80.08 (79.83-80.33)	76.04 (75.77-76.32)	81.23 (80.98-81.48)	78.91 (78.64-79.17)
Positive predictive value (%)	15.33 (14.91-15.75)	17.93 (17.45-18.41)	20.01 (19.49-20.53)	8.67 (8.32-9.03)	10.62 (10.18-11.06)	10.20 (9.79-10.60)
Negative predictive value (%)	98.41 (98.32-98.51)	98.49 (98.40-98.58)	98.77 (98.69-98.85)	99.38 (99.32-99.44)	99.34 (99.28-99.40)	99.57 (99.52-99.61)

^a^Values in parentheses indicate 95% CIs.

^b^Discrimination performance of the HR-B model and the HR-R model is based on C-statistics and that of the DL-R model is based on the area under the receiver operating characteristic curve.

^c^HR-B: hazard regression model with baseline data.

^d^HR-R: hazard regression model with repeated measurements.

^e^DL-R: deep learning model with repeated measurements.

**Table 4:** Comparison of the hazard regression model with repeated measurements and the deep learning model with repeated measurements using validation datasets from the National Health Insurance Service-Health Screening Cohort (40-79 years of age).

Performance index		All-cause dementia	Alzheimer dementia
		DL-R^a,b^ versus HR-R^c^	DL-R^a^ versus HR-R
**Discrimination**			
	Difference between AUCs^d^	0.034 (0.029-0.039)^e^	0.024 (0.018-0.031)^e^
	Absolute IDI^f^	0.334	0.423
	Relative IDI	3.200	5.351
**Reclassification**			
	Patients move to higher, n (%)	4163 (76.30)	2163 (86.42)
	Patients move to lower, n (%)	0 (0.00)	0 (0.00)
	Controls move to higher, n (%)	17,664 (19.52)	19,231 (21.25)
	Controls move to lower, n (%)	0 (0.00)	0 (0.00)
	NRI^g^ (%)	56.79^h^	65.17^h^

^a^NRIs were calculated to determine the improvement in the performance of each model to identify individuals whose risk of dementia was more than 50%.

^b^DL-R: deep learning model with repeated measurements.

^c^HR-R: hazard regression model with repeated measurements.

^d^AUC: area under the receiver operating characteristic curve.

^e^Difference between AUCs was significant with *P*<.001.

^f^IDI: integrated discrimination improvement.

^g^NRI: net reclassification improvement.

^h^NRI was significant with *P*<.001.

## Discussion

### Principal Findings

We investigated the accuracy of conventional hazards regression models and a deep learning RNN-LSTM model to predict all-cause dementia and Alzheimer dementia using a nationwide periodic health examination dataset. The deep learning algorithm showed better performance than the conventional hazards regression models. Previous studies have proposed methods to predict dementia using deep learning method together with multimodal imaging data, which can only be obtained through high-cost assessments such as neuroimaging (ie, magnetic resonance imaging [MRI] and positron emission tomography). To our knowledge, this is the first study of deep learning for the prediction of dementia using nationwide time-series health examination data.

We expected that the results of the RNN-LSTM model would reflect a complex relationship among the various risk factors. The RNN-LSTM models used a deep learning algorithm to achieve higher predictive accuracy than Cox regression models that used the same time-series data. The clinical implications of deep learning can be seen in a wide range of applications. In fact, some deep learning algorithms can distinguish normal dementia from Alzheimer dementia for diagnostic purposes or can predict the occurrence of Alzheimer dementia years in the future [18-20]. This study did not consider biomarkers of dementia, including neuroimaging results, and particularly biomarkers of Alzheimer dementia. It is costly to evaluate biomarkers of dementia and therefore not economical for everyone to undergo such expensive tests to predict dementia that has not yet occurred. By contrast, our deep learning model only requires data from routine health examinations, which can be obtained at a fraction of the cost of biomarker data. Our deep learning model can therefore be used for widespread screening to identify high-risk individuals who need further, more expensive tests such as genotyping, amyloid scanning, structural MRI, or neurocognitive testing. Used in such a way, our deep learning model based on risk factors from regular health examinations might improve the prediction of dementia among the undiagnosed population. Individuals who are identified as high risk by our predictive model can also receive medical counseling about preventive medicine to help prevent disease. Further studies are required to estimate the costs, benefits, and effectiveness of the use of our model to identify individuals at risk for dementia.

Our deep learning model showed good performance in screening out low-risk individuals. Although we did not provide statistical evidence that our model performed better for a younger population (aged 40-59 years) than for an older population (aged 60-79 years), the ability to accurately predict dementia in a younger population would be advantageous because it would help provide targeted prevention services to individuals at a younger age. In that sense, aggressive health management measures starting in midlife are crucial for preventing dementia.

A drawback of the deep learning model is that it cannot provide concrete recommendations to control specific risk factors. Although we ranked the risk factors in the DL-R model separately, the model does not explain how much particular risk factors affect the hazard ratio because of the nature of the hidden layer, which is considered a *black box* in neural network models [40]. By contrast, the HR-R model can show the magnitude of the risk for each factor, allowing specific guidelines to be given to reduce the effects of the most important risk factors. The individual risk levels identified by the HR-R model are important because control of specific risk factors might be necessary for certain undiagnosed individuals. Deep learning algorithms can be combined with conventional statistical methods such as the HR-R model to establish a special program to identify (1) individuals in the general population who are at risk for dementia and (2) lifestyle factors that should be modified to prevent dementia based on individual risk levels. It is difficult to predict and actively prevent dementia because of the multifactorial etiology of the disease. In countries where national health examinations are conducted, the use of our deep learning model might help to predict dementia and establish appropriate health policies. In the United Kingdom, the incidence of dementia is actually lower than previously predicted [41], suggesting that epidemiological investigations alone cannot accurately predict or respond to dementia.

To prevent dementia at the individual level, early identification and intervention in high-risk individuals are needed. Growing evidence indicates that individuals who maintain a healthy lifestyle and remain in good health, especially in midlife, can substantially reduce their risk of developing dementia [1-7]. Our findings that dementia can be predicted using simple clinical and lifestyle data suggest that dementia prevention strategies should focus on midlife health.

We also found that the SDs of some risk factors (ie, BMI, blood pressure, fasting glucose, and total cholesterol) were predictive of all-cause dementia and Alzheimer dementia; that is, the intraindividual variability of some risk factors influences the occurrence of dementia, which is consistent with the results of a previous study [42]. For instance, an individual with fluctuating body weight has a higher risk of developing dementia than an individual with stable body weight. Further research is needed to determine the relationship between dementia and variability in body weight or blood pressure.

### Limitations

Our study has some limitations. First, because we analyzed an established cohort that was not regularly tested for cognitive function, we could not include measurements of cognitive function in our predictive models. Nevertheless, according to a guideline of the South Korea National Health Insurance Review and Assessment Service, a cognitive enhancer may be prescribed to a patient with a Mini-Mental State Examination score of 26 or less out of 30 points, which corresponds to a diagnostic code for dementia. In addition, our models had higher predictive accuracy than the models used in some previous cohort studies that included measures of cognitive function [43-46]. Second, although we differentiated Alzheimer dementia from all-cause dementia on the basis of diagnostic codes, potential inaccuracies in diagnostic coding can occur in any study that uses medical claims data. Considering that our study was for predictive purposes, our results could identify significant cases that may require further specific and costly clinical evaluation such as genotyping, structural MRI, amyloid scanning, or neuropsychological testing. Although our model might currently be used to make policy decisions for dementia prevention, it might also be used for bedside applications in the future. It is not clear whether one-time health examination data are better than repeated measurements data for the prediction of dementia; therefore, future studies should compare the performance of dementia prediction models using each of those types of data. A third limitation of our study is that we could not measure the real onset of dementia. As the development of dementia (especially Alzheimer dementia) is insidious, the time of the first diagnosis by a clinician is usually delayed. A diagnosis of dementia in our study means that cognitive function was impaired enough to be diagnosed in a clinic. Thus, our result and definition of the time to event should be interpreted carefully. Finally, because the duration of follow-up was only 10 years, the deep learning algorithm might not have been sufficiently trained to accurately predict dementia in middle-aged individuals in the real world. When it becomes possible to do so, we will repeat our study using a follow-up of 20 to 30 years. Despite its limitations, our study had the advantage of using a large and relatively unbiased database, which is valuable in a public health context.

### Conclusions

A deep learning algorithm trained on nationwide periodic health examination data to predict dementia might be superior to specific biomarkers in terms of costs and benefits. Deep learning methods combined with conventional Cox hazards regression may provide useful information for the prediction and management of dementia. There is currently no curative treatment for all-cause dementia or Alzheimer dementia, but their early prediction in the general population can improve public health by facilitating prevention and early treatment. The data-driven, inductive approach of our models will contribute to efforts to tackle the global burden of dementia.

## Appendix

Multimedia Appendix 1 Characteristics of the development and the validation datasets by age group.

Multimedia Appendix 2 Iconography of some neural networks.

Multimedia Appendix 3 Constructing a predictive model in the recurrent neural network (RNN).

Multimedia Appendix 4 Variables used in each predictive model.

Multimedia Appendix 5 Hazard ratios for dementia risk factors in the Cox hazards regression model with baseline data (HR-B) from the development datasets from the National Health Insurance Service-Health Screening Cohort.

Multimedia Appendix 6 Hazard ratios for dementia risk factors in the Cox hazards regression model with repeated measurements (HR-R) from the development datasets from the National Health Insurance Service-Health Screening Cohort.

Multimedia Appendix 7 Ranking of the risk factors put into the deep learning model.

Multimedia Appendix 8 Comparison of model discrimination using the validation datasets from the National Health Insurance Service-Health Screening Cohort (40-59 and 60-79 years of age).

Multimedia Appendix 9 Comparison of area under receiver operating characteristics curve (AUC) between the hazard regression model with repeated measurements (HR-R) and the deep learning model with repeated measurements (DL-R) using the validation datasets from the National Health Insurance Service-Health Screening Cohort (40-79 years of age).

Multimedia Appendix 10 Calibration plots for each model by age group.

## Abbreviations

AUC: area under the receiver operating characteristic curve
BMI: body mass index
DBP: diastolic blood pressure
DL-R: deep learning model with repeated measurements
FCS: fully conditional specification
HR-B: Cox proportional hazards regression model with baseline data
HR-R: Cox hazards regression model with repeated measurements
LRP: layerwise relevance propagation
LSTM: long short-term memory
MI: multiple imputation
MRI: magnetic resonance imaging
NHIS: National Health Insurance Service
NHIS-HEALS: National Health Insurance Service-Health Screening Cohort
NRI: net reclassification improvement
RNN: recurrent neural network
SBP: systolic blood pressure
