# Supplementary material for: Cox Proportional Hazard Regression Versus a Deep Learning Algorithm in the Prediction of Dementia: An Analysis Based on Periodic Health Examination
Source: JMIR Med Inform. 2019 Aug 30;7(3):e13139. doi: 10.2196/13139 (PMC6743261; doi:10.2196/13139)
Supplement: Multimedia Appendix 1 [file medinform_v7i3e13139_app1.pdf]

Multimedia Appendix 1. Characteristics of the development and the validation datasets by age group.

(1) Characteristics of the development datasets of the National Health Insurance Service-Health Screening Cohort (40-59 years of age).

**Variable**	**All-cause dementia** (N = 10,902)		**Alzheimer's dementia** (N = 4,190)	
Duration of follow-up, years	9.34 ± 2.15		9.39 ± 2.13	
Number of periodic health examinations, n	5.25 ± 2.52		5.26 ± 2.52	
Age, years	50.06 ± 5.68		50.42 ± 5.68	
Gender (female), n (%)	5,252 (48.2)		2,076 (49.5)	
	**Baseline**	**Repeated measurement**[a]	**Baseline**	**Repeated measurement**[a]
Body mass index, kg/m^2	24.09 ± 2.97	24.08 ± 2.79	24.05 ± 3.02	24.03 ± 2.81
Systolic blood pressure, mmHg	126.27 ± 18.02	125.82 ± 12.85	126.14 ± 17.78	125.66 ± 12.75
Diastolic blood pressure, mmHg	79.78 ± 11.99	78.75 ± 8.12	79.57 ± 11.86	78.55 ± 8.00
Fasting plasma glucose, mg/dL	99.21 ± 38.02	101.23 ± 26.97	100.03 ± 40.47	101.31 ± 27.61
Total cholesterol, mg/dL	201.71 ± 39.96	200.37 ± 30.11	203.00 ± 40.15	201.30 ± 30.25
Smoking, n (%)	2,650 (24.3)	2,459 (22.6)	991 (23.7)	939 (22.4)
No exercise, n (%)	4,733 (43.4)	6,919 (63.5)	1,786 (42.6)	2,668 (63.7)
Cardiovascular disease, n (%)	877 (8.0)	5,241 (48.1)	311 (7.4)	1,969 (47.0)
Diabetes, n (%)	522 (4.8)	1,504 (13.8)	200 (4.8)	599 (14.3)
Hypertension, n (%)	793 (7.3)	3,497 (32.1)	316 (7.5)	1,323 (31.6)
Psychiatric disorder, n (%)	517 (4.7)	4,155 (38.1)	204 (4.9)	1,654 (39.5)
Neurological disorder, n (%)	1,145 (10.5)	6,669 (61.2)	462 (11.0)	2,581 (61.6)

[a]Variables indicate mean values and standard deviations during serial health examinations.

(2) Characteristics of the development datasets from the National Health Insurance Service-Health Screening Cohort (60-79 years of age).

**Variable**	**All-cause dementia** (N = 32,746)		**Alzheimer's dementia** (N = 15,834)	
Duration of follow-up, years	8.89 ± 2.33		8.99 ± 2.27	
Number of periodic health examinations, n	4.04 ± 1.55		4.08 ± 1.54	
Age, years	66.59 ± 5.06		66.70 ± 5.13	
Gender (female), n (%)	17,883 (54.6)		8,784 (55.5)	
	**Baseline**	**Repeated measurement**[a]	**Baseline**	**Repeated measurement**[a]
Body mass index, kg/m^2	23.90 ± 3.12	23.76 ± 2.98	23.88 ± 3.13	23.73 ± 2.98
Systolic blood pressure, mmHg	133.33 ± 19.39	132.07 ± 13.06	132.96 ± 19.36	131.77 ± 12.96
Diastolic blood pressure, mmHg	80.97 ± 11.85	79.57 ± 7.64	80.61 ± 11.73	79.36 ± 7.54
Fasting plasma glucose, mg/dL	102.72 ± 38.66	103.51 ± 26.62	102.72 ± 39.39	103.53 ± 26.49
Total cholesterol, mg/dL	202.84 ± 39.98	198.80 ± 31.35	202.66 ± 39.60	198.87 ± 31.30
Smoking, n (%)	5,435 (16.6)	4,793 (14.6)	2,581 (16.3)	2,260 (14.3)
No exercise, n (%)	10,725 (32.8)	16,054 (49.0)	5,147 (32.5)	7,701 (48.6)
Cardiovascular disease, n (%)	5,106 (15.6)	21,217 (64.8)	2,424 (15.3)	10,082 (63.7)
Diabetes, n (%)	2,612 (8.0)	6,362 (19.4)	1,262 (8.0)	3,072 (19.4)
Hypertension, n (%)	5,961 (18.2)	16,735 (51.1)	2,751 (17.4)	7,970 (50.3)
Psychiatric disorder, n (%)	1,973 (6.0)	14,586 (44.5)	950 (6.0)	7,101 (44.9)
Neurological disorder, n (%)	5,371 (16.4)	23,692 (72.4)	2,633 (16.6)	11,382 (71.9)

[a]Variables indicate mean values and standard deviations during serial health examinations.

(3) Characteristics of the validation datasets from the National Health Insurance Service-Health Screening Cohort (40-59 years of age).

Variable	All-cause dementia (N = 71,300)		Alzheimer's dementia (N = 70,519)	
Duration of follow-up, years	10.61 ± 1.11		10.64 ± 1.04	
Number of periodic health examinations, n	6.08 ± 2.68		6.11 ± 2.68	
Age, years	47.98 ± 5.39		47.93 ± 5.38	
Gender (female), n (%)	31,006 (43.5)		30,639 (43.4)	
	**Baseline**	**Repeated measurement**[a]	**Baseline**	**Repeated measurement**[a]
Body mass index, kg/m^2	24.04 ± 2.90	24.06 ± 2.74	24.03 ± 2.90	24.05 ± 2.74
Systolic blood pressure, mmHg	124.65 ± 17.05	124.46 ± 11.81	124.62 ± 17.05	124.41 ± 11.80
Diastolic blood pressure, mmHg	79.05 ± 11.64	78.15 ± 7.62	79.05 ± 11.66	78.13 ± 7.62
Fasting plasma glucose, mg/dL	96.65 ± 32.55	99.25 ± 21.51	96.54 ± 32.52	99.19 ± 21.38
Total cholesterol, mg/dL	199.61 ± 37.92	199.55 ± 28.80	199.49 ± 37.90	199.48 ± 28.78
Smoking, n (%)	18,714 (26.3)	16,768 (23.5)	18,478 (26.2)	16,551 (23.5)
No exercise, n (%)	32,677 (45.8)	49,778 (69.8)	32,434 (46.0)	49,379 (70.0)
Cardiovascular disease, n (%)	3,344 (4.7)	24,908 (34.9)	3,256 (4.6)	24,416 (34.6)
Diabetes, n (%)	2,083 (2.9)	8,516 (11.9)	1,997 (2.8)	8,333 (11.8)
Hypertension, n (%)	3,598 (5.1)	21,189 (29.7)	3,467 (4.9)	20,879 (29.6)
Psychiatric disorder, n (%)	2,022 (2.8)	18,679 (26.2)	1,979 (2.8)	18,299 (26.0)
Neurological disorder, n (%)	5,237 (7.4)	35,904 (50.4)	5,115 (7.3)	35,361 (50.1)
Event rate, n (%)	1,362 (1.9)		524 (0.7)	

[a]Variables indicate mean values and standard deviations during serial health examinations.

(4) Characteristics of the validation datasets from the National Health Insurance Service-Health Screening Cohort (60-79 years of age).

Variable	All-cause dementia (N = 24,642)		Alzheimer's dementia (N = 22,531)	
Duration of follow-up, years	9.75 ± 2.00		9.98 ± 1.82	
Number of periodic health examinations, n	4.41 ± 1.61		4.50 ± 1.60	
Age, years	65.69 ± 4.82		65.49 ± 4.74	
Gender (female), n (%)	12,751 (51.7)		11,445 (50.8)	
	**Baseline**	**Repeated measurement**[a]	**Baseline**	**Repeated measurement**[a]
Body mass index, kg/m^2	23.93 ± 3.07	23.80 ± 2.95	23.93 ± 3.06	23.80 ± 2.95
Systolic blood pressure, mmHg	132.67 ± 19.29	131.59 ± 12.58	132.57 ± 19.33	131.5 ± 12.52
Diastolic blood pressure, mmHg	80.78 ± 11.64	79.33 ± 7.36	80.8 ± 11.74	79.32 ± 7.33
Fasting plasma glucose, mg/dL	101.11 ± 35.99	102.46 ± 23.98	100.89 ± 34.13	102.24 ± 23.33
Total cholesterol, mg/dL	202.60 ± 39.26	198.14 ± 30.85	202.48 ± 38.9	197.86 ± 30.45
Smoking, n (%)	4,406 (17.9)	3,841 (15.6)	4,043 (17.9)	3,494 (15.5)
No exercise, n (%)	8,610 (34.9)	13,134 (53.3)	7,932 (35.2)	12,257 (54.4)
Cardiovascular disease, n (%)	3,308 (13.4)	14,960 (60.7)	2,900 (12.9)	13,519 (60.0)
Diabetes, n (%)	1,732 (7.0)	4,725 (19.2)	1,509 (6.7)	4,283 (19.0)
Hypertension, n (%)	4,049 (16.4)	12,786 (51.9)	3,626 (16.1)	11,682 (51.9)
Psychiatric disorder, n (%)	1,205 (4.9)	10,102 (41.0)	1,046 (4.6)	9,150 (40.6)
Neurological disorder, n (%)	3,593 (14.6)	17,058 (69.2)	3,164 (14.0)	15,457 (68.6)
Event rate, n (%)	4,094 (16.6)		1,980 (8.8)	

[a]Variables indicate mean values and standard deviations during serial health examinations.
