# Supplementary material for: Cox Proportional Hazard Regression Versus a Deep Learning Algorithm in the Prediction of Dementia: An Analysis Based on Periodic Health Examination
Source: JMIR Med Inform. 2019 Aug 30;7(3):e13139. doi: 10.2196/13139 (PMC6743261; doi:10.2196/13139)
Supplement: Multimedia Appendix 2 [file medinform_v7i3e13139_app2.pdf]

Multimedia Appendix 2. Iconography of some neural networks. (a) Vanilla (simple) feedforward neural network; (b) deep neural network; (c) vanilla (simple) recurrent neural network (RNN); (d) long short-term memory (LSTM).

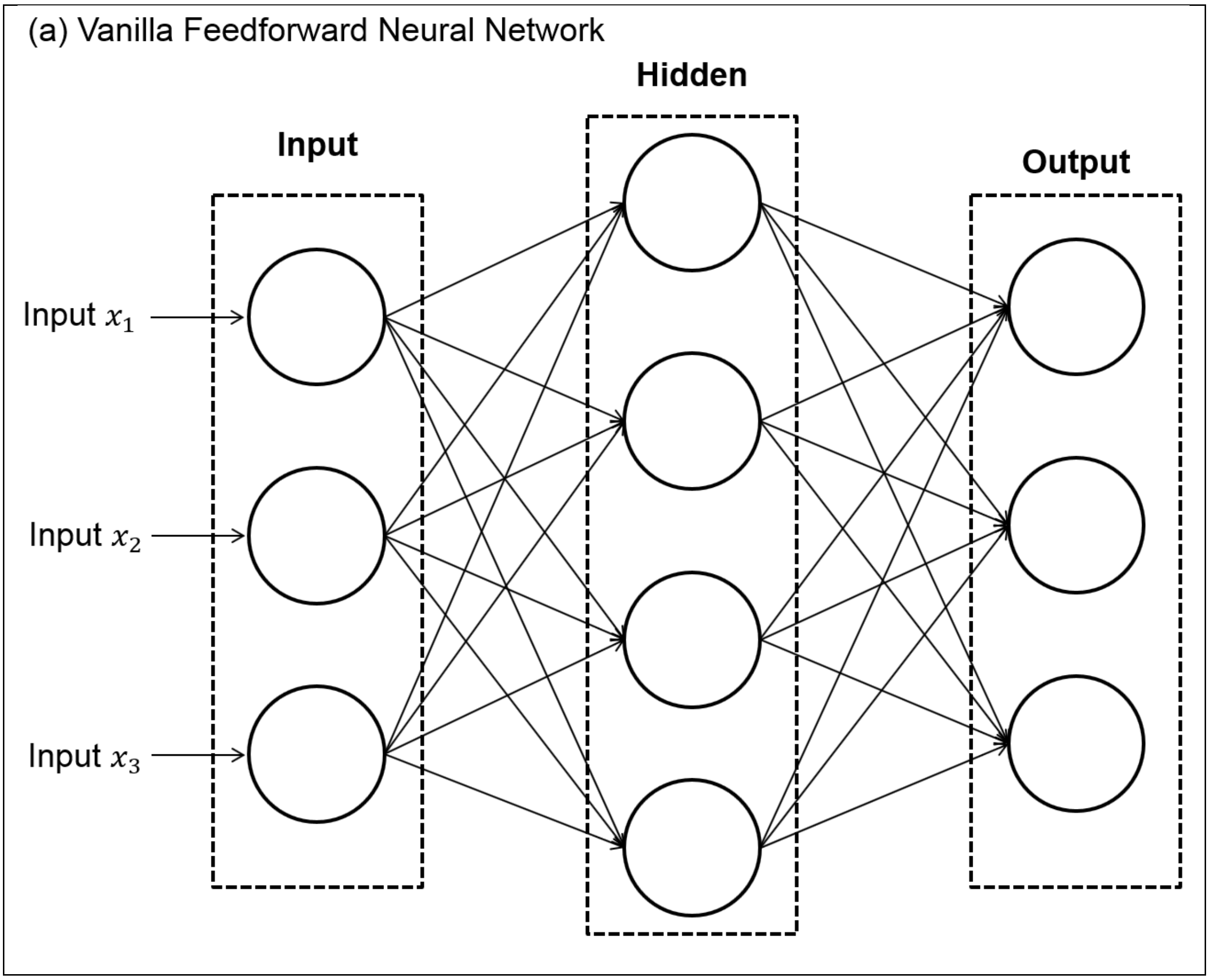

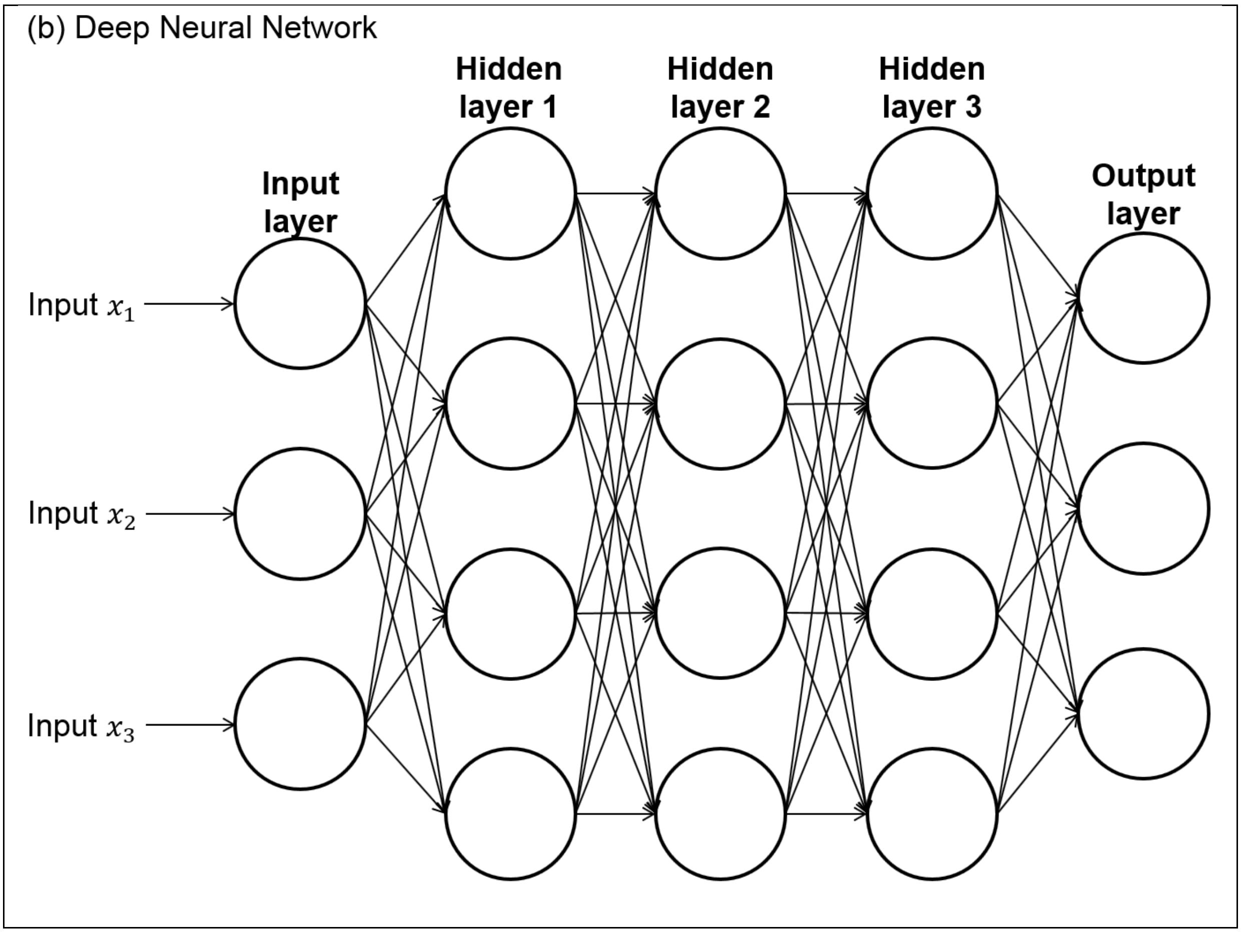

(b) Deep Neural Network
Hidden layer 1
Hidden layer 2
Hidden layer 3
Input layer
Output layer
Input x_1
Input x_2
Input x_3

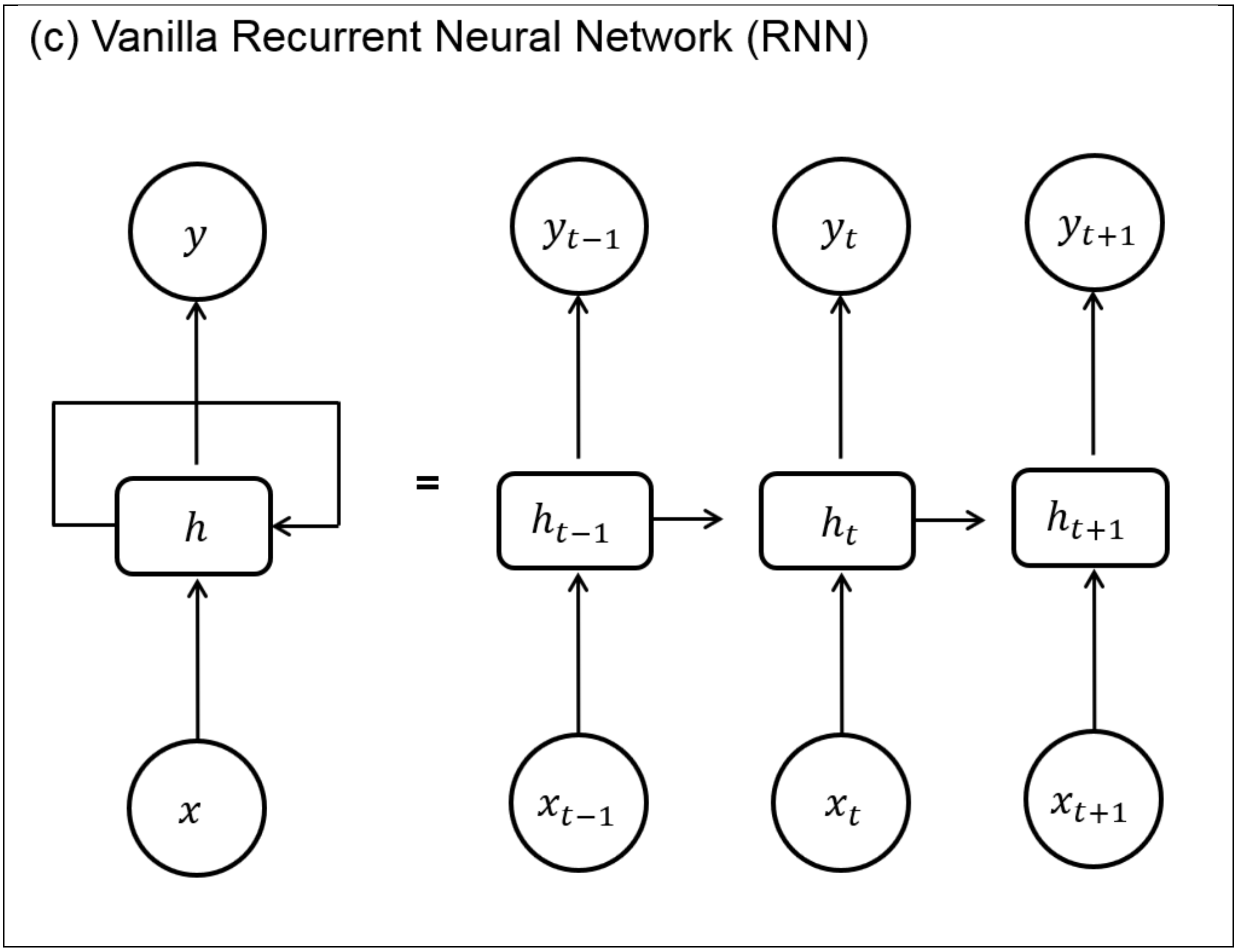
(c) Vanilla Recurrent Neural Network (RNN)
y
h
x
=
y_{t-1}
y_t
y_{t+1}
h_{t-1}
h_t
h_{t+1}
x_{t-1}
x_t
x_{t+1}

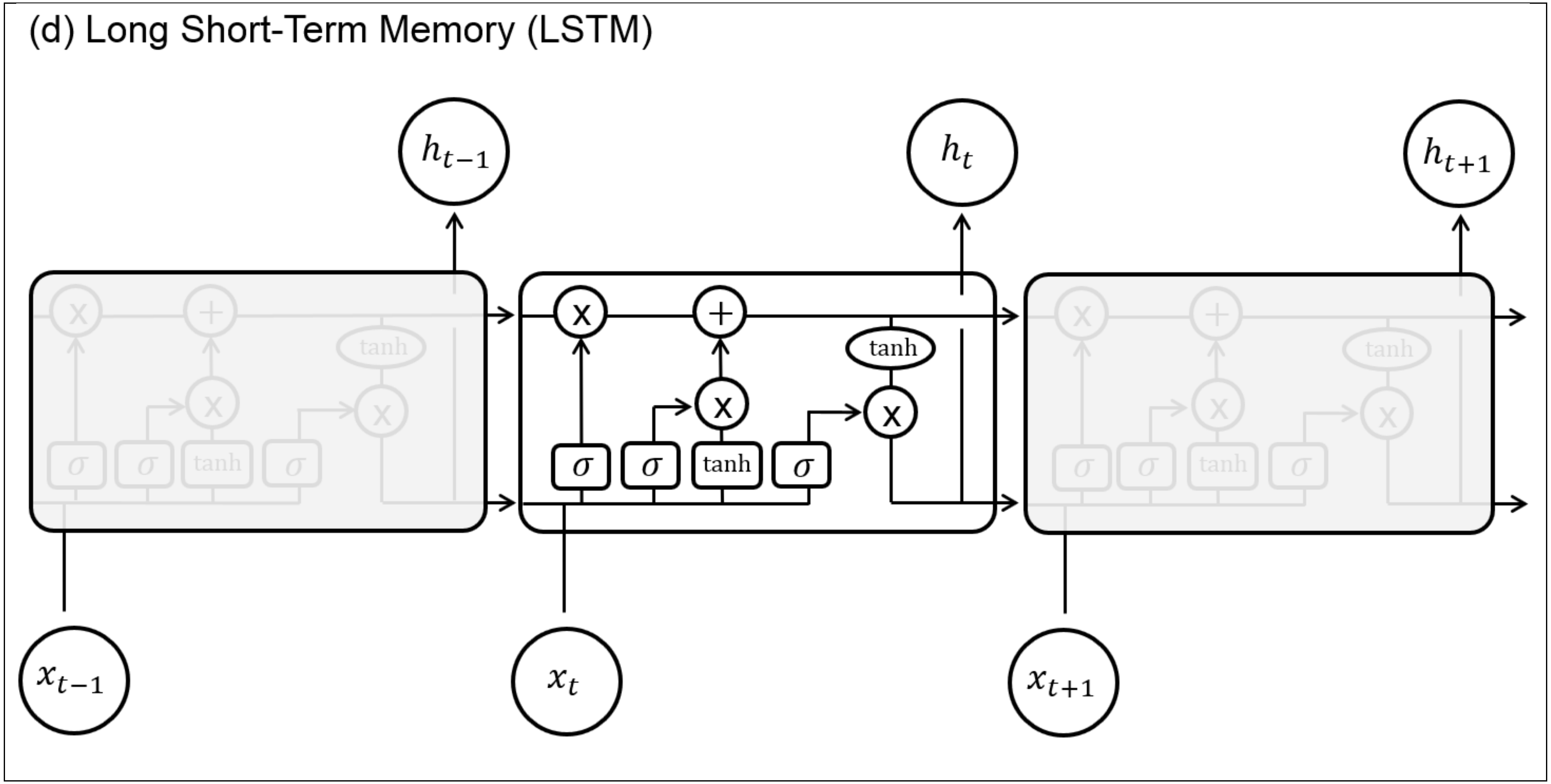

(d) Long Short-Term Memory (LSTM)
h_{t-1}
h_t
h_{t+1}
x
+
tanh
σ
σ
tanh
σ
x_{t-1}
x_t
x_{t+1}
