# Supplementary material for: Cox Proportional Hazard Regression Versus a Deep Learning Algorithm in the Prediction of Dementia: An Analysis Based on Periodic Health Examination
Source: JMIR Med Inform. 2019 Aug 30;7(3):e13139. doi: 10.2196/13139 (PMC6743261; doi:10.2196/13139)
Supplement: Multimedia Appendix 3 [file medinform_v7i3e13139_app3.pdf]

Multimedia Appendix 3. Constructing a predictive model in the recurrent neural network (RNN).

The learning sample set $\mathbb{S}$ is consisted of ordered pairs $(\mathbf{x}, \mathbf{y})$ with inputs and correct answers. Input x is an element of input set $\mathbb{X}$, whereas correct answer $\mathbf{y}$ is an element of correct answers set $\mathbb{Y}$. Here, $\mathbf{x}$ is consisted of serial data in the form of an individual's information. The T in input $\mathbf{x} = (\mathbf{x}_1, \dots, \mathbf{x}_\mathrm{t}, \dots, \mathbf{x}_\mathrm{T})$ represents the length of a sample (i.e. the number of events) and varies individually. A health examination record is consisted of an event; and, all events $\mathbf{x}_1, \dots, \mathbf{x}_\mathrm{T}$ for a person are in chronological order. x_t represents an event (examination record) at a specific time and is used as a vector with 9 features. The correct answer $\mathbf{y} = (\mathrm{y}_1, \dots, \mathrm{y}_\mathrm{K})$ has a Boolean value indicating whether or not dementia occurred in the past. The correct answer is one-hot encoded to be used as cross entropy in a loss function. K—the number of classes—is set as 2. Assuming the output of prediction model is $\hat{\mathrm{y}} = (\hat{\mathrm{y}}_1, \dots, \hat{\mathrm{y}}_K)$, cross entropy as shown in Equation 1 is used for our loss function.

$$\mathrm{E}(\mathrm{y}, \hat{\mathrm{y}}) = -\sum_{i=1}^{K} \lceil y_i \log(\hat{y}_i) + (1 - y_i)\log(1 - \hat{y}_i) \rceil \quad \text{(Equation 1)}$$

The output sequence for Long Short-Term Memory (LSTM) was $\mathbf{o} = (\mathbf{o}_1, \dots \mathbf{o}_\mathrm{t}, \dots, \mathbf{o}_\mathrm{T})$ where T was the event length being the same as the event length of x. The prediction result of an input sample provided the probability of dementia in the near future taken the occurrence of past event into account.

Only the last output $\mathbf{o}_\mathrm{T}$ among $\mathbf{o}_1, \dots \mathbf{o}_\mathrm{t}, \dots, \mathbf{o}_\mathrm{T}$ was used and reflected to an output z as shown in Equation 2. Here, $\mathrm{W} \in \mathbb{R}^{K \times H}$ was the parameter to be optimized and H was the number of hidden nodes in the last hidden layer. To calculate the probability of $\hat{y}_i$ from z, the Softmax function was used as shown in Equation 3.

$$\mathrm{z} = \mathrm{W}\mathbf{o}_\mathrm{T} \quad \text{(Equation 2)}$$

$$\hat{y}_\mathrm{i} = \frac{e^{z_i}}{\sum_{j=1}^{K} e^{z_j}} \quad \text{for } i = 1, \dots, K \quad \text{(Equation 3)}$$

LSTM had a memory cell with input, forget and output gates. Each LSTM unit uses the equations in Equation 4 which are commonly used in LSTM.

$$\mathrm{i_t} = \sigma(\mathrm{W}_{xi}\mathrm{x_t} + \mathrm{W}_{hi}\mathrm{h}_{t-1} + \mathrm{W}_{ci} \circ \mathrm{c}_{t-1} + b_i)$$

$$\mathrm{f_t} = \sigma\left(\mathrm{W}_{xf}\mathrm{x_t} + \mathrm{W}_{hf}\mathrm{h}_{t-1} + \mathrm{W}_{cf} \circ \mathrm{c}_{t-1} + b_f\right)$$

$$\mathrm{c_t} = f_{t\circ}c_{t-1} + \mathrm{i}_t \circ \tanh(\mathrm{W}_{xc}\mathrm{x}_t + \mathrm{W}_{hc}\mathrm{h}_{t-1} + b_c)$$

$$\mathrm{o_t} = \sigma(\mathrm{W}_{xo}\mathrm{x_t} + \mathrm{W}_{ho}\mathrm{h}_{t-1} + \mathrm{W}_{co} \circ \mathrm{c}_t + b_o)$$

$$\mathrm{h}_t = \mathrm{o}_t \circ \tanh(\mathrm{c}_t) \qquad \text{(Equation 4)}$$

σ was the logistic sigmoid function and i, f, o and c were respectively the *input gate*, *forget gate*, *output gate*, and *cell*. The subscripts in weight matrix above have an obvious meaning. For example, W_{hi} is the hidden-input gate matrix while W_{xo} being the input-output gate matrix. The bs are bias terms which are added for i, f, o and c equations. Let N be the number of LSTM blocks and M the number of inputs, $\mathrm{W}_i, \mathrm{W}_f, \mathrm{W}_c, \mathrm{W}_o \in \mathbb{R}^{N\times M}$
