# Supplementary material for: Cox Proportional Hazard Regression Versus a Deep Learning Algorithm in the Prediction of Dementia: An Analysis Based on Periodic Health Examination
Source: JMIR Med Inform. 2019 Aug 30;7(3):e13139. doi: 10.2196/13139 (PMC6743261; doi:10.2196/13139)
Supplement: Multimedia Appendix 4 [file medinform_v7i3e13139_app4.pdf]

Multimedia Appendix 4. Variables used in each predictive model.

Model	Variables
HR-B[a]	Age, gender, body mass index, systolic blood pressure, diastolic blood pressure, fasting plasma glucose, total cholesterol, smoking, exercise status, cardiovascular disease, diabetes, hypertension, psychiatric disorder, and neurological disorder
HR-R[b]	Age, body mass index, systolic blood pressure, diastolic blood pressure, fasting plasma glucose, total cholesterol (mean, standard deviation, minimum, and maximum), gender, smoking, exercise status, cardiovascular disease, diabetes, hypertension, psychiatric disorder, and neurological disorder (mean, standard deviation)
DL-R[c]	Date of each health examination, age, gender, body mass index, systolic blood pressure, diastolic blood pressure, fasting plasma glucose, total cholesterol, smoking, exercise status, cardiovascular disease, diabetes, hypertension, psychiatric disorder, and neurological disorder at each health examination

[a]HR-B: hazard regression model with baseline data only.

[b]HR-R: hazard regression model with repeated measurements.

[c]DL-R: deep learning model with repeated measurements.
