# Supplementary material for: Cox Proportional Hazard Regression Versus a Deep Learning Algorithm in the Prediction of Dementia: An Analysis Based on Periodic Health Examination
Source: JMIR Med Inform. 2019 Aug 30;7(3):e13139. doi: 10.2196/13139 (PMC6743261; doi:10.2196/13139)
Supplement: Multimedia Appendix 5 [file medinform_v7i3e13139_app5.pdf]

Multimedia Appendix 5. Hazard ratios for dementia risk factors in the Cox hazards regression model with baseline data (HR-B) from the development datasets from the National Health Insurance Service-Health Screening Cohort.

	All-cause dementia[a]						Alzheimer's dementia[a]					
	40–79 years of age		40–59 years of age		60–79 years of age		40–79 years of age		40–59 years of age		60–79 years of age	
**Variable**	**HR[b] (95% CI)[c]**	***P***	**HR[b] (95% CI)[c]**	***P***	**HR[b] (95% CI)[c]**	***P***	**HR[b] (95% CI)[c]**	***P***	**HR[b] (95% CI)[c]**	***P***	**HR[b] (95% CI)[c]**	***P***
Age per 5 years	1.51 (1.50, 1.52)	< .001	1.60 (1.56, 1.64)	< .001	1.48 (1.46, 1.50)	< .001	1.57 (1.55, 1.58)	< .001	1.74 (1.67, 1.83)	< .001	1.50 (1.47, 1.53)	< .001
Female	1.14 (1.10, 1.17)	< .001	1.17 (1.10, 1.25)	< .001	1.21 (1.17, 1.25)	< .001	1.22 (1.16, 1.27)	< .001	1.28 (1.16, 1.42)	< .001	1.25 (1.19, 1.31)	< .001
Body mass index per kg/m^2	1.00 (0.99, 1.00)	.439	1.01 (1.00, 1.02)	.139	0.99 (0.99, 1.00)	.001	1.00 (0.99, 1.00)	.454	1.00 (0.99, 1.02)	.611	0.99 (0.98, 1.00)	.012
Systolic blood pressure per 10 mmHg	1.02 (1.01, 1.03)	< .001	1.04 (1.02, 1.07)	.001	1.01 (1.00, 1.02)	.099	1.03 (1.01, 1.04)	.001	1.04 (1.00, 1.07)	.082	1.02 (1.01, 1.04)	.007
Diastolic blood pressure per 5 mmHg	1.00 (0.99, 1.01)	.763	0.98 (0.97, 1.00)	.053	1.00 (0.99, 1.01)	.774	0.99 (0.98, 1.01)	.226	0.99 (0.96, 1.02)	.389	0.99 (0.98, 1.00)	.111
Fasting plasma glucose per 10 mg/dL	1.01 (1.00, 1.01)	< .001	1.00 (1.00, 1.01)	.320	1.01 (1.01, 1.02)	< .001	1.01 (1.00, 1.01)	.004	1.00 (0.99, 1.02)	.509	1.01 (1.00, 1.01)	.003
Total cholesterol per 10 mg/dL	1.00 (0.99, 1.00)	.232	1.00 (0.99, 1.01)	.846	1.00 (0.99, 1.00)	.081	1.00 (1.00, 1.00)	.946	0.99 (0.98, 1.01)	.293	1.00 (0.99, 1.00)	.243
Smoking	1.05 (1.01, 1.09)	.014	1.10 (1.03, 1.19)	.009	1.10 (1.05, 1.15)	< .001	1.04 (0.99, 1.11)	.151	1.09 (0.96, 1.23)	.180	1.05 (0.99, 1.13)	.125
No exercise	1.07 (1.03, 1.10)	< .001	1.08 (1.02, 1.14)	.009	1.09 (1.05, 1.13)	< .001	1.02 (0.97, 1.06)	.492	1.08 (0.98, 1.18)	.112	1.04 (0.99, 1.09)	.102
Cardiovascular disease	1.33 (1.26, 1.40)	< .001	1.43 (1.29, 1.60)	< .001	1.09 (1.06, 1.13)	< .001	1.16 (1.10, 1.23)	< .001	1.24 (1.07, 1.44)	.004	1.08 (1.02, 1.13)	.003
Diabetes	1.25 (1.21, 1.30)	< .001	1.33 (1.23, 1.45)	< .001	1.07 (1.03, 1.11)	.001	1.28 (1.19, 1.37)	< .001	1.12 (0.93, 1.35)	.244	1.38 (1.28, 1.50)	< .001
Hypertension	1.25 (1.21, 1.30)	< .001	1.31 (1.20, 1.43)	< .001	1.16 (1.12, 1.20)	< .001	1.00 (0.94, 1.05)	.894	1.06 (0.92, 1.23)	.429	0.99 (0.93, 1.05)	.671
Psychiatric disorder	1.26 (1.19, 1.32)	< .001	1.21 (1.08, 1.36)	.001	1.26 (1.19, 1.34)	< .001	1.30 (1.21, 1.41)	< .001	1.62 (1.36, 1.91)	< .001	1.17 (1.11, 1.22)	< .001
Neurological disorder	1.03 (0.99, 1.07)	.109	1.09 (0.99, 1.20)	.070	1.03 (0.99, 1.07)	.200	1.27 (1.20, 1.34)	< .001	1.32 (1.17, 1.51)	< .001	1.03 (0.98, 1.09)	.302

[a]Variables in the parenthesis indicate 95% confidence intervals.

[b]HR, hazard ratio; [c]CI, confidence interval.
