# Supplementary material for: Cox Proportional Hazard Regression Versus a Deep Learning Algorithm in the Prediction of Dementia: An Analysis Based on Periodic Health Examination
Source: JMIR Med Inform. 2019 Aug 30;7(3):e13139. doi: 10.2196/13139 (PMC6743261; doi:10.2196/13139)
Supplement: Multimedia Appendix 6 [file medinform_v7i3e13139_app6.pdf]

Multimedia Appendix 6. Hazard ratios for dementia risk factors in the Cox hazards regression model with repeated measurements (HR-R) from the development datasets from the National Health Insurance Service-Health Screening Cohort.

Variable		All-cause dementia[a]						Alzheimer's dementia[a]					
		40–79 years of age		40–59 years of age		60–79 years of age		40–79 years of age		40–59 years of age		60–79 years of age	
		HR[b] (95% CI)[c]	*P*	HR[b] (95% CI)[c]	*P*	HR[b] (95% CI)[c]	*P*	HR[b] (95% CI)[c]	*P*	HR[b] (95% CI)[c]	*P*	HR[b] (95% CI)[c]	*P*
Age per 5 years		1.39 (1.38, 1.40)	< .001	1.43 (1.39, 1.47)	< .001	1.33 (1.31, 1.35)	< .001	1.46 (1.44, 1.47)	< .001	1.54 (1.47, 1.62)	< .001	1.37 (1.34, 1.40)	< .001
Female		1.01 (0.98, 1.04)	.663	1.04 (0.98, 1.12)	.202	1.02 (0.99, 1.06)	.213	1.06 (1.02, 1.12)	.009	1.16 (1.04, 1.29)	.009	1.07 (1.01, 1.13)	.013
Body mass index per kg/m^2	Mean	1.06 (1.01, 1.11)	.030	1.04 (0.94, 1.15)	.469	1.12 (1.05, 1.19)	< .001	0.99 (0.92, 1.07)	.823	0.96 (0.83, 1.12)	.604	1.09 (1.00, 1.18)	.061
	SD[d]	1.35 (1.28, 1.43)	< .001	1.61 (1.41, 1.84)	< .001	1.36 (1.28, 1.45)	< .001	1.29 (1.19, 1.40)	< .001	1.66 (1.38, 2.00)	< .001	1.32 (1.20, 1.44)	< .001
Systolic blood pressure per 10 mmHg	Mean	1.00 (0.95, 1.05)	.997	1.01 (0.91, 1.13)	.804	1.01 (0.95, 1.08)	.661	1.02 (0.94, 1.10)	.678	1.05 (0.88, 1.25)	.582	1.00 (0.92, 1.09)	.954
	SD[d]	1.03 (1.03, 1.04)	< .001	1.03 (1.02, 1.05)	< .001	1.04 (1.03, 1.05)	< .001	1.03 (1.02, 1.04)	< .001	1.03 (1.01, 1.06)	.003	1.04 (1.03, 1.06)	< .001
Diastolic blood pressure per 5 mmHg	Mean	1.02 (0.99, 1.06)	.227	0.96 (0.89, 1.04)	.292	1.01 (0.97, 1.06)	.563	0.99 (0.94, 1.05)	.784	0.93 (0.82, 1.04)	.211	0.99 (0.93, 1.06)	.832
	SD[d]	1.05 (1.04, 1.06)	< .001	1.08 (1.05, 1.10)	< .001	1.06 (1.05, 1.07)	< .001	1.04 (1.02, 1.05)	< .001	1.07 (1.03, 1.10)	< .001	1.04 (1.02, 1.06)	< .001
Fasting plasma glucose per 10 mg/dL	Mean	0.95 (0.93, 0.98)	< .001	0.98 (0.93, 1.02)	.247	0.96 (0.93, 0.99)	.009	0.96 (0.92, 1.00)	.027	0.99 (0.91, 1.07)	.729	0.93 (0.89, 0.98)	.002
	SD[d]	1.00 (1.00, 1.01)	.060	1.00 (0.99, 1.00)	.166	1.01 (1.00, 1.01)	.004	1.00 (1.00, 1.01)	.446	1.00 (0.99, 1.01)	.474	1.00 (1.00, 1.01)	.200
Total cholesterol per 10 mg/dL	Mean	0.99 (0.96, 1.01)	.292	0.98 (0.94, 1.03)	.445	1.00 (0.97, 1.02)	.720	1.00 (0.96, 1.03)	.767	1.02 (0.94, 1.09)	.660	1.00 (0.97, 1.05)	.831
	SD[d]	1.03 (1.02, 1.03)	< .001	1.03 (1.02, 1.03)	< .001	1.03 (1.02, 1.03)	< .001	1.03 (1.03, 1.03)	< .001	1.02 (1.01, 1.03)	< .001	1.03 (1.02, 1.03)	< .001
Smoking	Mean	0.99 (0.95, 1.03)	.620	1.04 (0.96, 1.13)	.295	0.99 (0.94, 1.05)	.824	1.04 (0.97, 1.12)	.241	1.06 (0.93, 1.22)	.400	0.96 (0.88, 1.04)	.267
	SD[d]	1.03 (0.95, 1.11)	.513	0.94 (0.81, 1.09)	.443	1.12 (1.02, 1.22)	.022	0.87 (0.77, 0.98)	.020	0.97 (0.76, 1.26)	.844	1.06 (0.92, 1.27)	.405
No exercise	Mean	1.26 (1.22, 1.30)	< .001	1.24 (1.17, 1.31)	< .001	1.33 (1.29, 1.37)	< .001	1.22 (1.17, 1.27)	< .001	1.25 (1.14, 1.37)	< .001	1.26 (1.20, 1.32)	< .001
	SD[d]	0.96 (0.92, 1.02)	.163	0.94 (0.85, 1.05)	.273	0.90 (0.85, 0.96)	.001	0.96 (0.89, 1.04)	.286	0.89 (0.75, 1.06)	.176	0.93 (0.85, 1.02)	.112
Cardiovascular disease		1.28 (1.24, 1.32)	< .001	1.44 (1.35, 1.52)	< .001	1.18 (1.14, 1.22)	< .001	1.21 (1.16, 1.27)	< .001	1.28 (1.16, 1.40)	< .001	1.12 (1.07, 1.18)	< .001
Diabetes		1.12 (1.07, 1.16)	< .001	1.01 (0.92, 1.10)	.918	1.10 (1.05, 1.15)	< .001	1.08 (1.01, 1.14)	.017	1.04 (0.89, 1.20)	.645	1.21 (1.13, 1.29)	< .001
Hypertension		0.91 (0.88, 0.94)	< .001	0.87 (0.81, 0.93)	< .001	0.91 (0.88, 0.94)	< .001	0.86 (0.82, 0.90)	< .001	0.86 (0.77, 0.96)	.006	0.83 (0.79, 0.87)	< .001
Psychiatric disorder		1.16 (1.13, 1.19)	< .001	1.30 (1.22, 1.38)	< .001	1.11 (1.07, 1.14)	< .001	1.21 (1.16, 1.26)	< .001	1.49 (1.36, 1.64)	< .001	1.18 (1.12, 1.23)	< .001
Neurological disorder		1.20 (1.16, 1.24)	< .001	1.33 (1.25, 1.43)	< .001	1.09 (1.05, 1.13)	< .001	1.17 (1.12, 1.23)	< .001	1.21 (1.09, 1.34)	< .001	1.07 (1.02, 1.14)	.010

[a]Variables in the parenthesis indicate 95% confidence intervals.

[b]HR: hazard ratio; [c]CI: confidence interval; [d]SD: standard deviation.
