# Supplementary material for: Cox Proportional Hazard Regression Versus a Deep Learning Algorithm in the Prediction of Dementia: An Analysis Based on Periodic Health Examination
Source: JMIR Med Inform. 2019 Aug 30;7(3):e13139. doi: 10.2196/13139 (PMC6743261; doi:10.2196/13139)
Supplement: Multimedia Appendix 7 [file medinform_v7i3e13139_app7.pdf]

Multimedia Appendix 7. Ranking of the risk factors put into the deep learning model.

Rank	All-cause dementia			Alzheimer's dementia		
	Total	Age		Total	Age	
		40–59	60–79		40–59	60–79
1	Gender	Gender	Gender	Gender	Age	Gender
2	Age	Exercise	Age	Age	Fasting plasma glucose	Exercise
3	Exercise	Smoking	Neurological disorder	Neurological disorder	Body mass index	Neurological disorder
4	Smoking	Age	Cardiovascular disease	Cardiovascular disease	Systolic blood pressure	Cardiovascular disease
5	Cardiovascular disease	Neurological disorder	Hypertension	Systolic blood pressure	Diastolic blood pressure	Psychiatric disorder
6	Neurological disorder	Diabetes	Total cholesterol	Psychiatric disorder	Exercise	Hypertension
7	Hypertension	Total cholesterol	Diabetes	Diastolic blood pressure	Psychiatric disorder	Fasting plasma glucose
8	Diabetes	Systolic blood pressure	Smoking	Smoking	Neurological disorder	Body mass index
9	Psychiatric disorder	Hypertension	Psychiatric disorder	Hypertension	Cardiovascular disease	Total cholesterol
10	Diastolic blood pressure	Cardiovascular disease	Systolic blood pressure	Fasting plasma glucose	Diabetes	Diabetes
11	Body mass index	Diastolic blood pressure	Exercise	Body mass index	Hypertension	Smoking
12	Fasting plasma glucose	Psychiatric disorder	Diastolic blood pressure	Exercise	Gender	Diastolic blood pressure
13	Systolic blood pressure	Body mass index	Fasting plasma glucose	Total cholesterol	Smoking	Systolic blood pressure
14	Total cholesterol	Fasting plasma glucose	Body mass index	Diabetes	Total cholesterol	Age
