# Supplementary material for: Cox Proportional Hazard Regression Versus a Deep Learning Algorithm in the Prediction of Dementia: An Analysis Based on Periodic Health Examination
Source: JMIR Med Inform. 2019 Aug 30;7(3):e13139. doi: 10.2196/13139 (PMC6743261; doi:10.2196/13139)
Supplement: Multimedia Appendix 8 [file medinform_v7i3e13139_app8.pdf]

Multimedia Appendix 8. Comparison of model discrimination using the validation datasets from the National Health Insurance Service-Health Screening Cohort (40-59 and 60-79 years of age).

Age		All-cause dementia[a,b]			Alzheimer's dementia[a,b]		
		**HR-B[c]**	**HR-R[d]**	**DL-R[e]**	**HR-B[c]**	**HR-R[d]**	**DL-R[e]**
40–59	Discrimination (Performance)	0.75 (0.73, 0.76)	0.80 (0.79, 0.82)	0.86 (0.86, 0.86)	0.76 (0.73, 0.78)	0.82 (0.80, 0.85)	0.86 (0.86, 0.87)
	Sensitivity (%)	69.60 (67.16, 72.05)	71.29 (68.89, 73.69)	78.78 (76.61, 80.96)	70.23 (66.31, 74.14)	80.34 (76.94, 83.75)	76.53 (72.90, 80.16)
	Specificity (%)	67.80 (67.46, 68.15)	72.49 (72.16, 72.83)	78.50 (78.19, 78.80)	70.65 (70.31, 70.98)	65.74 (65.39, 66.09)	78.74 (78.44, 79.04)
	Accuracy (%)	67.84 (67.50, 68.18)	72.47 (72.14, 72.80)	78.50 (78.20, 78.81)	70.64 (70.31, 70.98)	65.85 (65.50, 66.20)	78.73 (78.42, 79.03)
	Positive predictive value (%)	4.04 (3.79, 4.29)	4.81 (4.51, 5.10)	6.66 (6.28, 7.05)	1.76 (1.58, 1.94)	1.73 (1.56, 1.89)	2.62 (2.37, 2.88)
	Negative predictive value (%)	99.14 (99.05, 99.22)	99.23 (99.16, 99.31)	99.48 (99.42, 99.54)	99.69 (99.64, 99.73)	99.78 (99.73, 99.82)	99.78 (99.74, 99.82)
60–79	Discrimination (Performance)	0.67 (0.66, 0.68)	0.76 (0.75, 0.77)	0.80 (0.79, 0.80)	0.70 (0.69, 0.71)	0.77 (0.76, 0.78)	0.79 (0.79, 0.79)
	Sensitivity (%)	69.65 (67.93, 70.76)	74.67 (73.34, 76.00)	69.74 (68.33, 71.14)	63.99 (61.88, 66.10)	72.48 (70.51, 74.44)	74.65 (72.73, 76.56)
	Specificity (%)	55.45 (54.77, 56.13)	61.20 (60.54, 61.57)	76.85 (76.27, 77.43)	65.48 (64.53, 66.13)	64.80 (64.15, 65.45)	70.33 (69.70, 70.95)
	Accuracy (%)	57.76 (57.14, 58.37)	63.44 (62.84, 64.04)	75.67 (75.13, 76.20)	65.35 (64.72, 65.97)	65.47 (64.85, 66.10)	70.71 (70.11, 71.30)
	Positive predictive value (%)	23.67 (22.91, 24.43)	27.72 (26.88, 28.55)	37.51 (36.42, 38.60)	15.15 (14.38, 15.92)	16.55 (15.77, 17.34)	19.51 (18.62, 20.40)
	Negative predictive value (%)	90.08 (89.56, 90.60)	92.38 (91.94, 92.83)	92.73 (92.34, 93.12)	94.97 (94.61, 95.33)	96.07 (95.74, 96.39)	96.64 (96.35, 96.93)

[a]Values in parentheses indicate 95% confidence intervals.

[b]Discrimination of HR-B and HR-R indicates C-statistics. The performance of DL-R indicates the area under the receiver-operating characteristics curve.

[c]HR-B, hazard regression model with baseline data only; [d]HR-R, hazard regression model with repeated measurements; [e]DL-R, deep learning model with repeated measurements.
