# Supplementary material for: Cox Proportional Hazard Regression Versus a Deep Learning Algorithm in the Prediction of Dementia: An Analysis Based on Periodic Health Examination
Source: JMIR Med Inform. 2019 Aug 30;7(3):e13139. doi: 10.2196/13139 (PMC6743261; doi:10.2196/13139)
Supplement: Multimedia Appendix 9 [file medinform_v7i3e13139_app9.pdf]

Multimedia Appendix 9. Comparison of area under receiver operating characteristics curve (AUC) between the hazard regression model with repeated measurements (HR-R) and the deep learning model with repeated measurements (DL-R) using the validation datasets from the National Health Insurance Service-Health Screening Cohort (40-79 years of age).

	HR-R[a,b]	DL-R[c]
All-cause dementia	0.862 (0.857–0.866)	0.896 (0.895–0.896)
Alzheimer's dementia	0.882 (0.876–0.888)	0.906 (0.906–0.907)

[a]To calculate the difference of performance between deep learning model with repeated measurements (DL-R) and hazard regression model with repeated measurements (HR-R), the performance of HR-R converted to the area under receiver-operating characteristics curve (AUC).

[b]HR-R, hazard regression model with repeated measurements; [c]DL-R, deep learning model with repeated measurements.
