# Supplementary material for: Cox Proportional Hazard Regression Versus a Deep Learning Algorithm in the Prediction of Dementia: An Analysis Based on Periodic Health Examination
Source: JMIR Med Inform. 2019 Aug 30;7(3):e13139. doi: 10.2196/13139 (PMC6743261; doi:10.2196/13139)
Supplement: Multimedia Appendix 10 [file medinform_v7i3e13139_app10.pdf]

Multimedia Appendix 10. Calibration plots for each model by age group.

(1) Calibration plots for each model for all-cause dementia by age group (40-59 and 60-79 years of age). HR-B: hazard regression model with baseline data only; HR-R: hazard regression model with repeated measurements; DL-R: deep learning model with repeated measurements.

<table>
<tr><td>Category</td><td colspan="2">All-cause dementia</td></tr>
<tr><td>Age group</td><td>40–59</td><td>60–79</td></tr>
<tr><td>HR-B</td><td>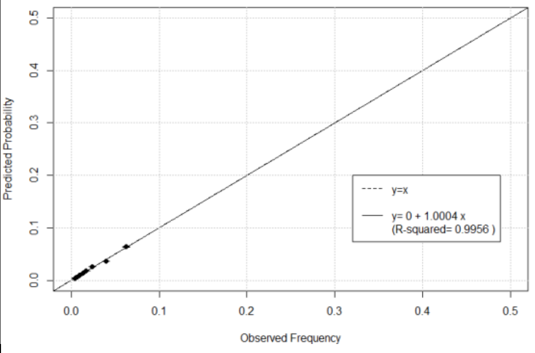
</td><td>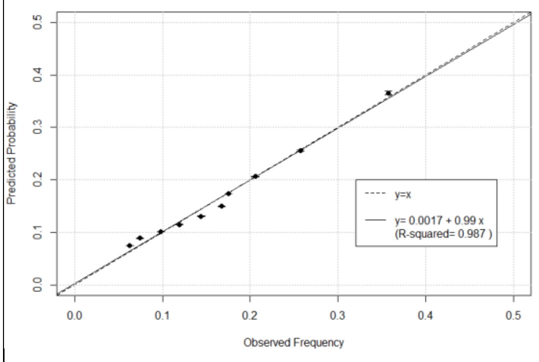
</td></tr>
<tr><td>HR-R</td><td>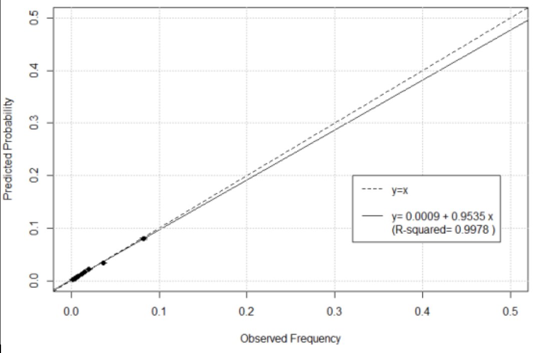
</td><td>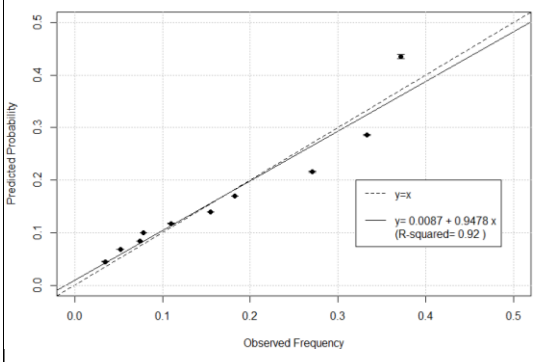
</td></tr>
<tr><td>DL-R</td><td>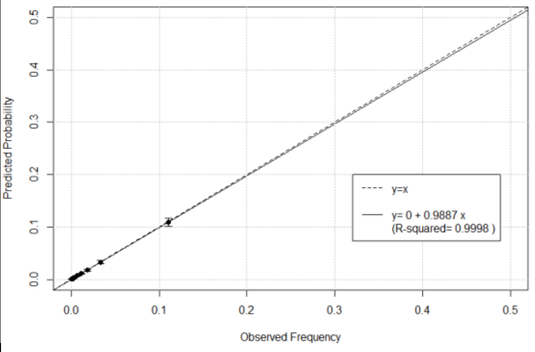
</td><td>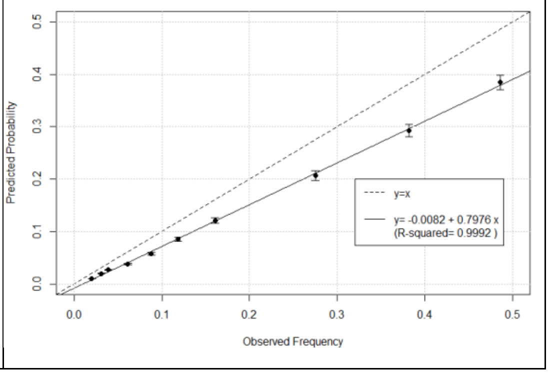
</td></tr>
</table>

(2) Calibration plots for each model for Alzheimer's dementia by age group (40-59 and 60-79 years of age). HR-B: hazard regression model with baseline data only; HR-R: hazard regression model with repeated measurements; DL-R: deep learning model with repeated measurements.

<table>
<tr><td>Category</td><td colspan="2">Alzheimer's dementia</td></tr>
<tr><td>Age group</td><td>40–59</td><td>60–79</td></tr>
<tr><td>HR-B</td><td>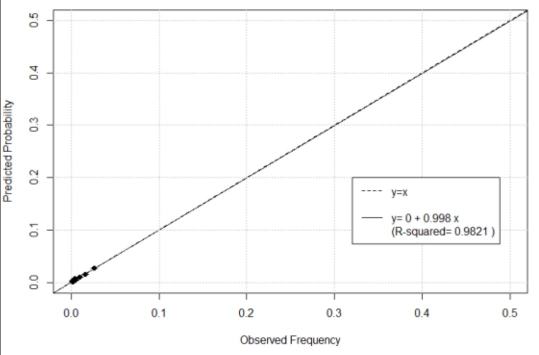
</td><td>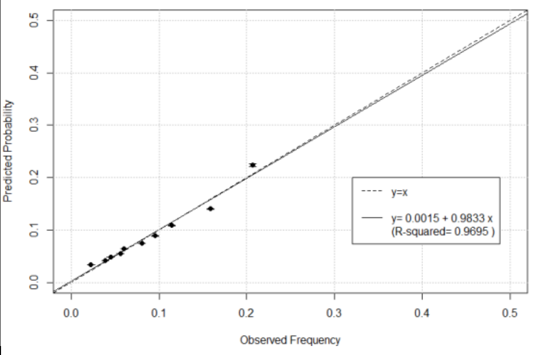
</td></tr>
<tr><td>HR-R</td><td>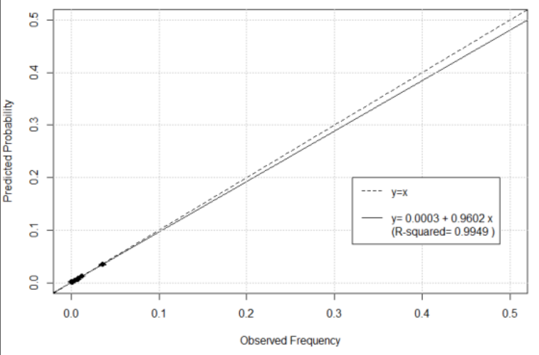
</td><td>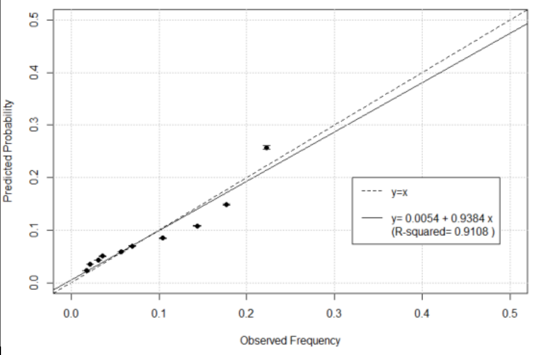
</td></tr>
<tr><td>DL-R</td><td>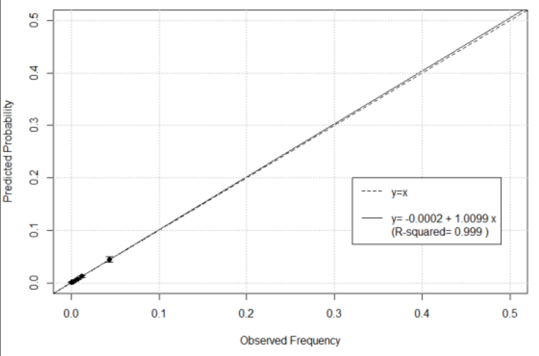
</td><td>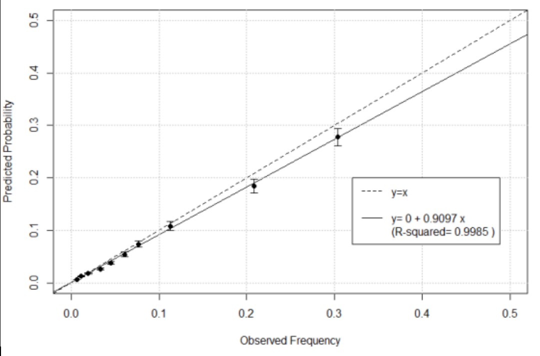
</td></tr>
</table>
